# Supplementary material for: Ocean acidification disrupts the biomineralization process in the oyster Crassostrea virginica via intracellular calcium signaling dysregulation
Source: Commun Biol. 2026 Mar 17;9:607. doi: 10.1038/s42003-026-09861-y (PMC13144384; doi:10.1038/s42003-026-09861-y)
Supplement: Supplementary file 1 — Supplementary Information [file 42003_2026_9861_MOESM1_ESM.pdf]

Title: Ocean acidification disrupts the biomineralization process in the oyster *Crassostrea virginica* via intracellular calcium signaling dysregulation

Chi Huang[1], Joseph Matt[2,3], Christopher Hollenbeck[2,3], Leisha Martin[2], Wei Xu[1*]

[1]Department of Veterinary Physiology and Pharmacology, College of Veterinary Medicine & Biomedical Science, Texas A&M University, College Station, Texas, 77843.

[2]Department of Life Sciences, College of Science, Texas A&M University - Corpus Christi, Corpus Christi, Texas, 78412.

[3]Corpus Christi Research and Extension Center, Texas A&M AgriLife Research, Corpus Christi, Texas, 78406.

*Corresponding author: Wei Xu, 402 Raymond Stotzer Pkwy, College Station, Texas, 77843.
E-mail: wxu1@tamu.edu

Supplementary Information

Supplementary Table 1. Formulation of solutions and growth medium used in this study. * All balanced salt solutions and growth medium were adjusted with HCl and NaOH.

Solution	Formulation	Reference
Ca^{2+} and Mg^{2+} free artificial seawater solution (CMFSS)	0.8 g/L KCL<br>25.5g/L NaCl<br>3.0 g/L Na_2PO_4<br>3.0 g/L D-Glucose (Dextrose) anhydrous,<br>2.86 g/L HEPES<br>pH = 7.7	[1]
Oyster cell culture medium	Leibovitz's L-15 Powder<br>NaCl 12.5 g/L<br>D-Glucose 6.24 g/L<br>$MgSO_4$ 3.16 g/L<br>KCl 0.344 g/L<br>$NaHCO_3$ 0.192 g/L<br>HEPES 2.86g/L<br>5.68 g/L $MgCl_2 \bullet 6H_2O$<br>1.48 g/L $CaCl_2 \bullet 2H_2O$<br>500 µL/L MEM non-essential amino acids<br>500 µL/L sodium pyruvate<br>1.75 g/L -tocopherol-acetate<br>1 mg/L Hydrocortisone<br>Insulin, Transferrin, and Sodium Selenit 500 µL/L<br>pH = 7.8	[1]

Supplementary Table 2. Ocean acidification treatment groups across *C. virginica* larval stage transitions.

Age of development	Treatment Hours	Developmental Stage	Density of larvae	Sample size per replicate
12 hours old	24	Trochophore stage	20 oysters /ml	~14,000
48 hours old	48	D-shaped stage	20 oysters /ml	~14,000
10 days old	48	Umbonal stage	2.5 oysters /ml	~1,750
20 days old	48	Pediveliger stage	1.5 oysters /ml	~1,050

Supplementary Table 3. Candidate genes and related primers for qPCR analysis

Target gene	Accession # of *C. virginica*	Homologous species (Accession #)	Identities (%)	E-value	Sequence	Reference
Cv-ef1α	BG624869.1	*C. virginica* (BG624869.1)	100	0	F: 5’-ATCAACTTCCACTGGCCATC-3‘<br>R: 5’-TTTTCCCATCTCAGCTGCTT-3‘	[2]
Cv-CaM	MG029431	*C. virginica* (MG029431)	100	0	F: 5’-ACAGATGAAGAGGTAGATGAAATGA-3‘<br>R: 5’-GCTATACAAGCAGTTGCCATTC-3‘	[1]
Cv-CaN	XM_022461887	*P. fucata* (EU797510)	99	0	F: 5’-TTGCTCACTCACCCTTAATCTG-3’<br>R: 5’-GCTGTGCTGCTTTCCATTTC-3’	[3]
Cv-Nacrein	XM_022487278.1	*P. fucata* (BAA11940.1)	31.33	$3e^{-42}$	F: 5’-GATGATACTGAGGAGGCCAAAG-3’<br>R: 5’-GTCCACAACATTCTCTGGTATAGG-3’	[4]
Cv-Pif97	XM_022443581	*M. gigas* (AFT63505.1)	72.46	0	F: 5’-AGACCTTAAGATTTGCCCAGATAG-3’<br>R: 5’-CGACTCCAGGCATCATACATT-3’	[5]
Cv-Tyr	XM_022479257.1	*M. gigas* (AGZ15753.1)	91.95	0	F: 5’-GGGACCATTCGCTCGATTTA-3’<br>R: 5’-GTAGCCGTCGATGTAGTTATGG-3’	[6]
Cv-Chits	XM_022473493	*A. rigida* (AAY86556.1)	46.61	0	F: 5’-CCGGGTGTTTCAGTTTGTTTAG-3’<br>R: 5’-GAATGTTTCGGGTGCATGTG-3’	[7]

Supplementary Table 4. Summary of the carbonate system parameter of C. virginica mantle cell culture medium and *in vivo* experiment used seawater. Average measured parameters: pH, salinity, pCO_2, and temperature. Average calculated parameters: bicarbonate ion concentration (HCO_3^-), carbonate (CO_3^{2-}), saturation state ($\Omega_{calcite}$), and aragonite saturation state ($\Omega_{aragonite}$), and dissolved inorganic carbon (DIC). The estimated carbonate chemistry parameter above were calculated by R package seacarb v3.3.2 [8] using the measured pH, pCO_2, temperature, and salinity. n is the number of observations. SD represents standard deviation.

Measured Parameters	Cell Culture medium	Cell Culture medium	Cell Culture medium	Seawater	Seawater
	Ambient Air pCO_2: 425 ppm (n = 3)	1.5% pCO_2 15000 ppm (n = 8)	2.5% pCO_2 25000 ppm (n = 3)	Present-day levels pCO_2: 425 ppm (n = 4)	Year 2100 levels pCO_2: 1000 ppm (n = 4)
pH ± SD	7.55±0.00577	7.37±0.0493	7.19±0.0603	7.88±0.0465	7.30±0.118
Temp ± SD (℃)	28.0 ±0.49	28.0±0.1	28.0±0.3	23.0±0.23	24.5±0.33
Sal± SD (psu)	25±0.13	24±0.2	25±0.4	37±0.816	34.8±0.5
Calculated Parameters					
HCO_3^- ± SD (uM)	568.29±7.58	13352.32±1503.30	14683.20±67.38	1347.26±138.96	847.59±212.97
CO_3^{2-} ± SD (μM)	19.01±0.51	300.39±67.38	218.31±58.68	105.80±20.46	18.52±8.59
ΩAr ± SD	0.32±0.0088	5.09±1.14	3.70±0.99	1.64±0.32	0.29±0.13
ΩCa ± SD	0.50±0.0132	7.90±1.77	5.74±1.54	2.33±0.78	0.44±0.20
DIC ± SD (μM)	599.04±8.09	14067.61±1570.53	15592.99±2068.56	1465.92±159.37	896.38±211.61

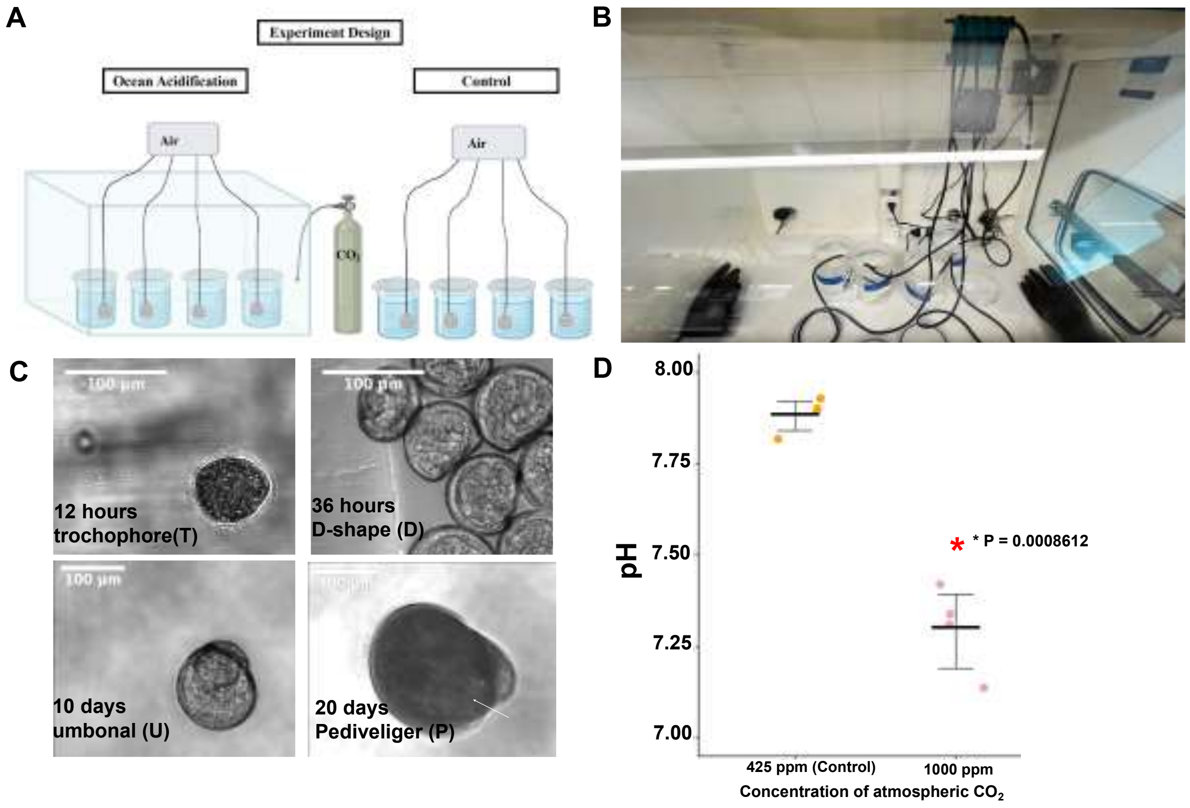

Supplementary Figure 1. Treatment strategies for Ocean acidification in oyster larvae. (A) The setup for testing oyster larvae development under simulated ocean acidification at the lab condition (Created in BioRender, https://BioRender.com/v5lvnmw). Four replicate beakers will be used for sample collection for morphology and qPCR analyses. (B) A treatment group of oyster larvae under a mimic acidification environment in a glove box. Ocean acidification condition = 1000$\pm$150 ppm atmospheric CO_2 concentration and control = 425$\pm$150 ppm atmospheric CO_2 concentration. (C) Light micrographs of *C. virginica* larvae incubated for 12 hours (trochophore stage), 48 hours (D-shape stage), 10 days (umbonal stage), and 20 days (pediveliger or eyed larvae). White arrow: “Eye” of eyed larvae. During the trochophore stage, the oyster embryo develops a hair-like structure called “cilia”, enabling its locomotion in the water column. In the D-shaped stage, a two-shell structure and an organ for movement and feeding begin to form. Subsequently, in the umbonal stage, the oyster larvae develop a hinge between the two shells. Following the umbonal stage, a locomotion-related structure will shortly be developed in preparation for permanent attachment to a hard surface in the pediveliger stage *[9–11]*. (D) Changes in filtered seawater pH for *C. virginica* larval development between Control and acidification treatment group (n = 3-4 beakers of seawater). *$P < 0.05$, Student's t-test was used for statistical test. Error bar: 95% confidence interval of the mean.

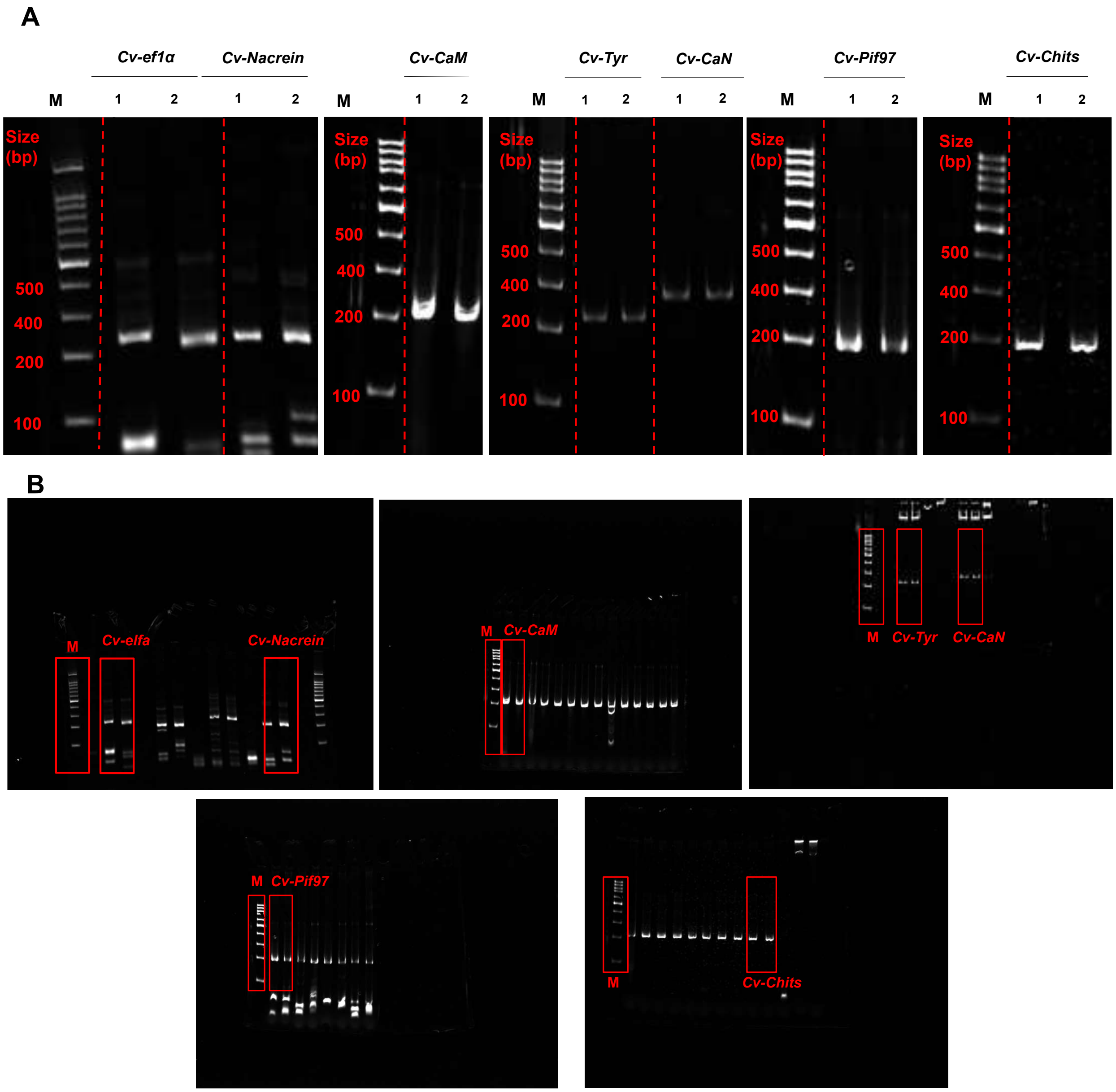

Supplementary Figure 2. *Crassostrea virginica* specific PCR primers for genetic markers were analyzed with qPCR and PAGE-TBE gel electrophoresis. (A) PCR products of *Cv-ef1α*, *Cv-Nacrein*, *Cv-CaM*, *Cv-Tyr*, *Cv-CaN*, *Cv-Pif97*, and *Cv-Chits* were detected in *C. virginica* mantle cells. M: DNA marker. The red dashed lines indicated the separation between different sets of gene markers. (B) Original uncropped PAGE-TBE gel electrophoresis images.

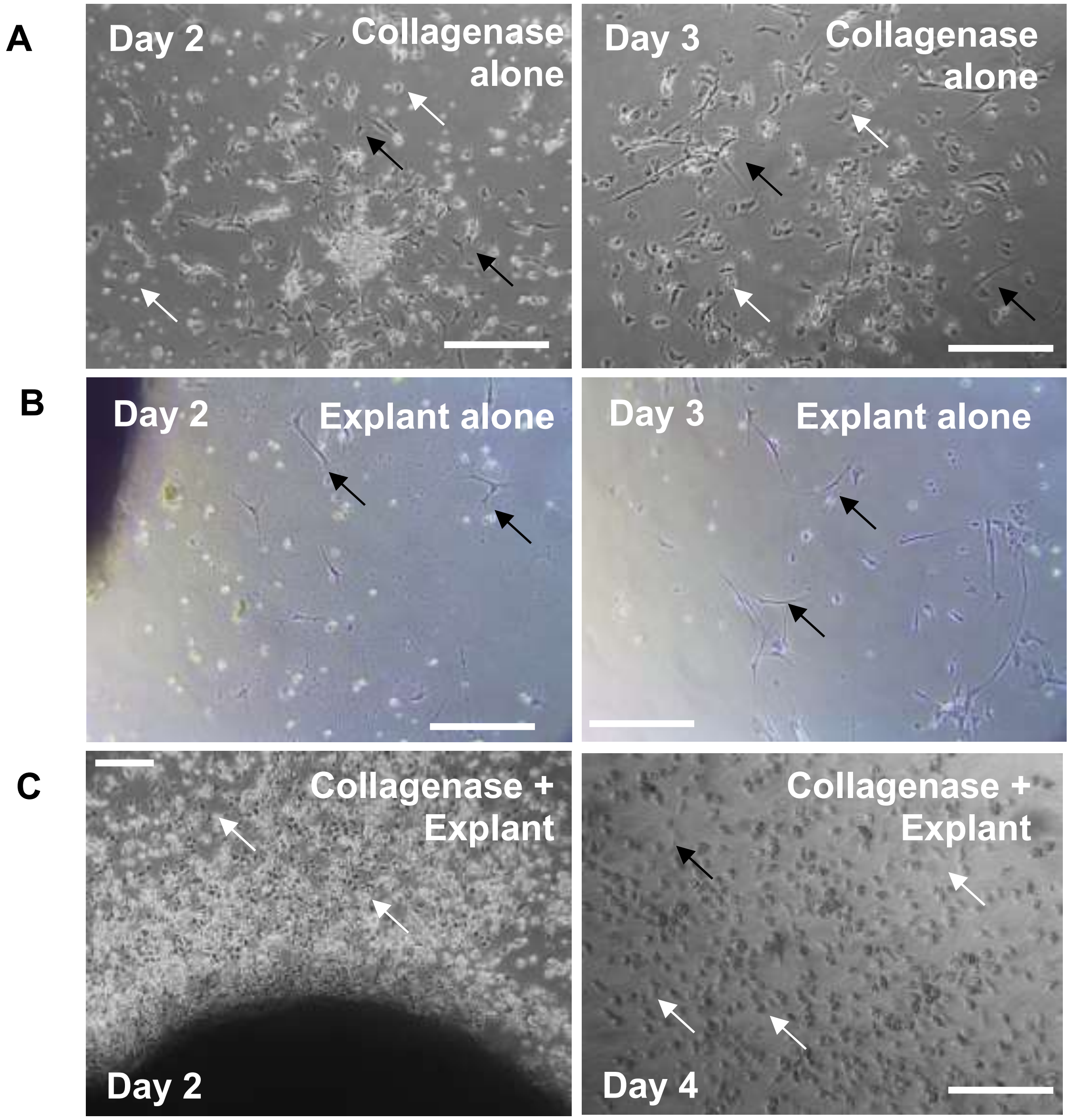

Supplementary Figure 3. Cell population within different preparations in primary mantle cell culture of *C. virginica*. (A) Dissociated mantle cells yield from collagenase alone. A large mixture of fibroblast- and epithelial-like cells. (B) Mantle explant culture without collagenase treatment. Fibroblast-like cells migrated from the explant. (C) Collagenase-treated explant. Most of the migrated cell shapes are closed to the epithelial cells. Black arrow: Fibroblast-like cell. White arrow: Epithelial-like cell. White bar: 100 μm.

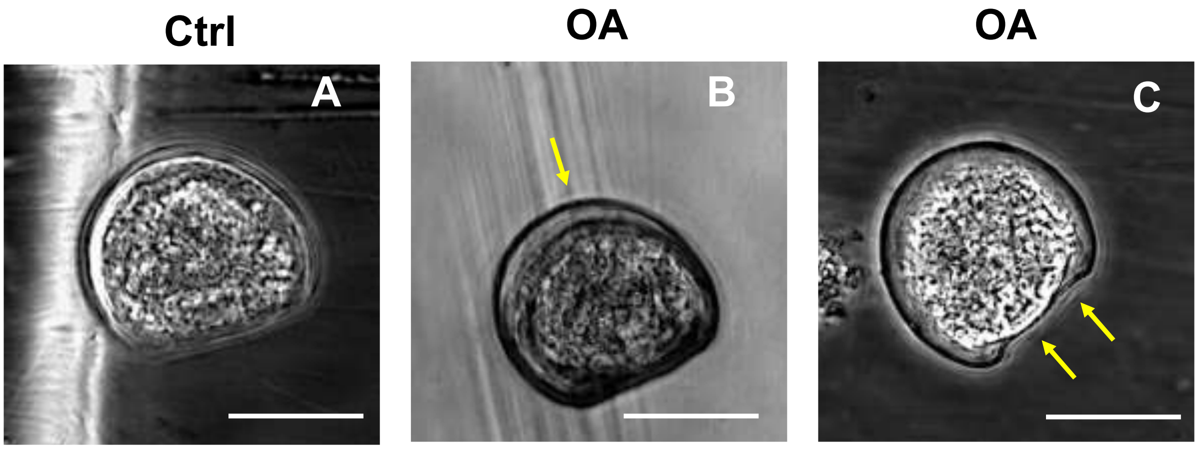

Supplementary Figure 4. OA condition alters early development of D-shape oyster larvae. (A) Representative image of a normally developed D-shaped shell at 36 hours post-fertilization in the control group. (B–C) Abnormal D-shaped shells observed at 36 hours post-fertilization under OA conditions, characterized by a protruding mantle along the shell margin (B) and a “concave” shell hinge (C). Yellow arrow: shell deformities. White scale bar: 100 μm.

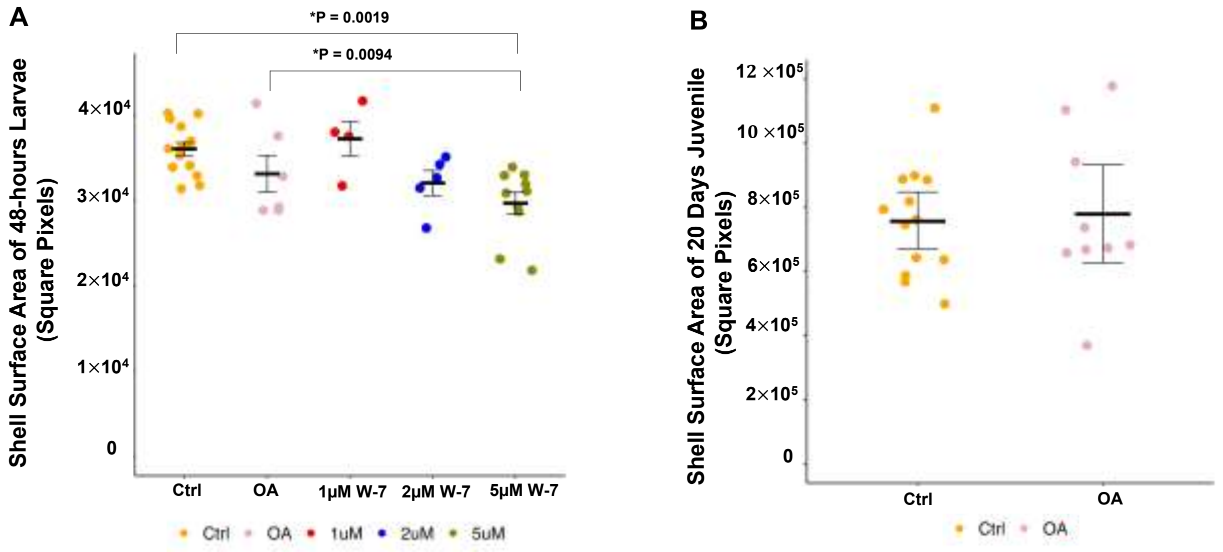

Supplementary Figure 5. Comparisons of average shell surface area of C. virginica among treatments for simulating ocean acidification (OA) or W-7 exposure (n = 4-16 oysters per treatment). (A) Average shell surface area of 48-hour D-shape larvae after 24 hours of OA or W-7 treatments. (B) Average shell surface area of 20-day-old veliger juveniles after two days of exposure to 1000ppm CO_2. Error bars represent 95% confidence intervals. *P < 0.05, one-way ANOVA followed by Westfall's method for multiple comparison.

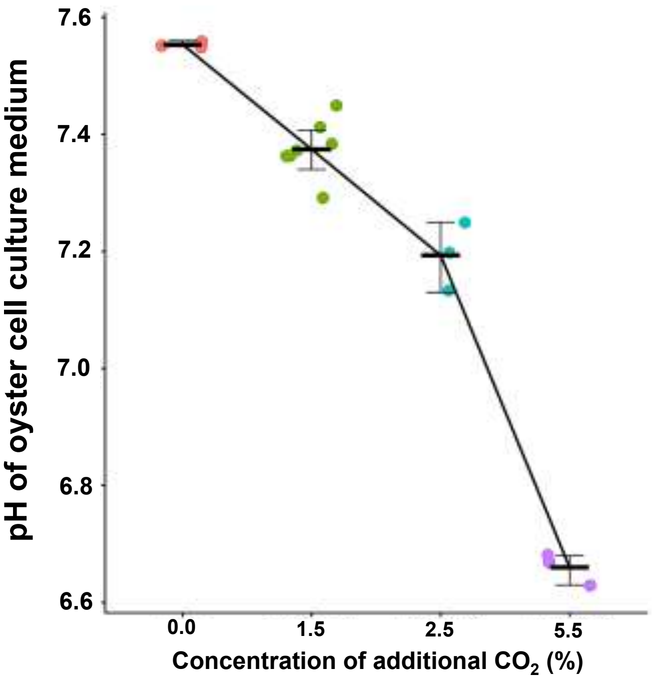

Supplementary Figure 6. Changes of oyster cell culture medium pH in response to increased atmospheric CO_2 after 48 hours (n = 3-6 culture flasks). Error bar: 95% confidence interval of the mean.

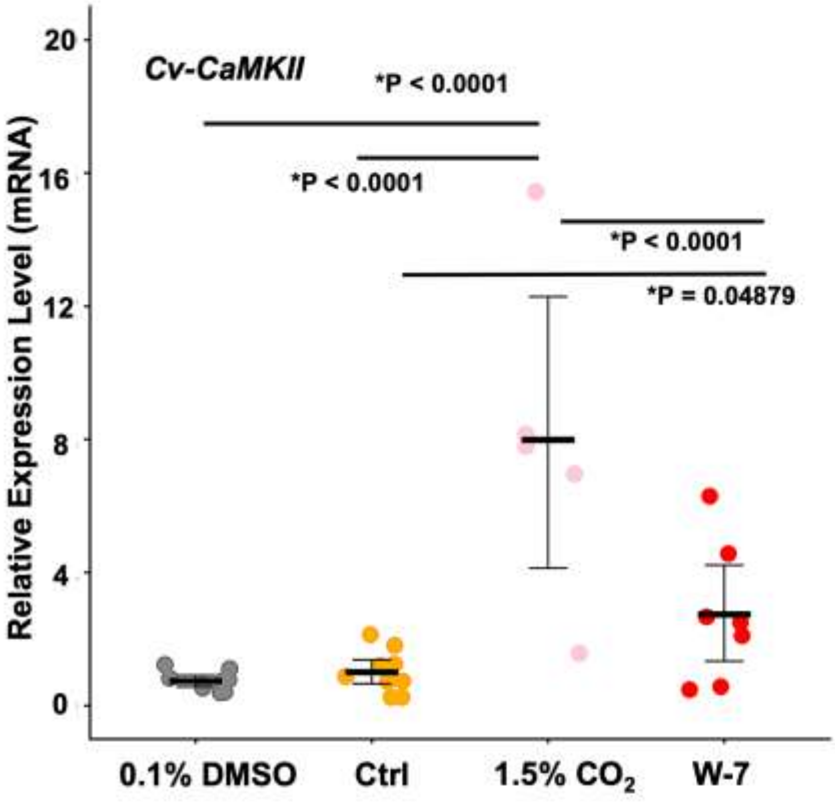

Supplementary Figure 7. Expression of Ca^{2+}/CaM dependent protein kinase II (CaMKII) in *C. virginica* mantel cells among treatment groups of 0.1% DMSO, Control, 1.5% CO_2 exposure, and 25 μM W-7 at mRNA level (n = 6-8 oysters). The significant difference between the two groups (P < 0.05) was demonstrated by different letters over the column. One-way ANOVA followed by Westfall's method for multiple comparison. Error bar: 95% confidence interval of the mean.

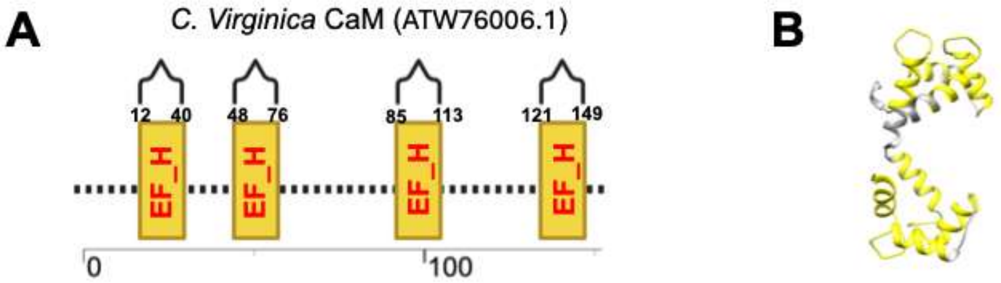

C

```
Crassostrea virginica   MADQLTEEQIAEFKEAFSLFDKDGDGTITTKELGTVMRSLGQNPTEAELQDMINEVDADGNGTIDFPEFLTMMARKMKDT 80
Pinctada fucata         MADQLTEEQIAEFKEAFSLFDKDGDGTITTKELGTVMRSLGQNPTEAELQDMINEVDADGNGTIDFPEFLTMMARKMKDT 80
Haliotis discus         MTEQMTQEQISEFKEAFGLFDKDGDGSITTSELGTVMRSLGQEPSQTELQNMIKEIDVDGNGLIEFPEFLAMMAKRMKS- 79
Drosophila melanogaster MADQLTEEQIAEFKEAFSLFDKDGDGTITTKELGTVMRSLGQNPTEAELQDMINEVDADGNGTIDFPEFLTMMARKMKDT 80
Homo sapiens            MADQLTEEQIAEFKEAFSLFDKDGDGTITTKELGTVMRSLGQNPTEAELQDMINEVDADGNGTIDFPEFLTMMARKMKDT 80

Crassostrea virginica   DSEEEIREAFRVFDKDGNGFISAAELRHVMTNLGEKLTDEEVDEMIREADIDGDGQVNYEEFVAMMTSK--- 149
Pinctada fucata         DSEEEIREAFRVFDKDGNGFISAAELRHVMTNLGEKLTDEEVDEMIREADIDGDGQVNYEEFVKMMMSKEFH 152
Haliotis discus         DADDEMREAFRVFDKDGNGFISAAELRHVMTNLGEKLTDEEVDDMIKEADTNGDGQVDYDEFVRMMKNQ--- 148
Drosophila melanogaster DSEEEIREAFRVFDKDGNGFISAAELRHVMTNLGEKLTDEEVDEMIREADIDGDGQVNYEEFVTMMTSK--- 149
Homo sapiens            DSEEEIREAFRVFDKDGNGYISAAELRHVMTNLGEKLTDEEVDEMIREADIDGDGQVNYEEFVQMMTAK--- 149
```

Supplementary Figure 8. Prediction of functional domains and 3D folding structures of *C. vriginica* calmodulin (CaM, Accession# ATW76006.1). (A) Four calcium-binding domains, E-F hands (EF_H), were predicted using the simple modular architecture research tool (SMART) (Schultz et al., 1998; Letunic et al., 2015). (B) 3D structure of *C. virginica* CaM was predicted using ColabFold https://colab.research.google.com/github/sokrypton/ColabFold/blob/main/AlphaFold2.ipynb (Kim et al., 2025). Yellow: EF_H domain (C) Multiple amino acid sequence alignment of *C.virginica* CaM with other CaM across taxa, including *Pinctada fucata* (bivalve, Accession# AAQ20043.1), *Haliotidis discus* (gastropod, Accession# QBS36239.1), *Drosophila melanogaster* (arthropod, Accession# NP_001246276.1), and *Homo sapiens* (vertebrate, Accession# NP_001316851.1) via Sequence Manupulation Suite https://www.bioinformatics.org/sms2/reference.html (Stothard, 2000).

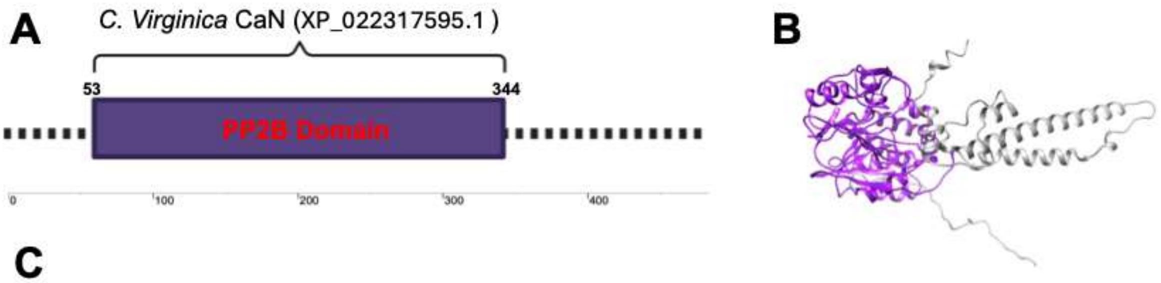

C

```
Crassostrea virginica   ------------------------------------------------------------------------MATT 4
Pinctada fucata         ------------------------------------------------------------------------MATT 4
Haliotis discus         ------------------------------------------------------------------------MATT 4
Drosophila melanogaster MSSPAAQSNSSSSQSQSAAQQQQQQNQKANVNNTHDNKNAAATTGTAAGSGSGGAAGSAGTQQQGQGGTGTSSGPSSPTK 80
Homo sapiens            ---------------------------------------------------------------------MSEPKAI 7

Crassostrea virginica   ETKLSTTDRVVKSVAFPPNHRLSMNEVFDA-KGKPKPDVLKQHFIMEGRVSEDVALRLINEGAALLRQEKTMIDIEAPVT 83
Pinctada fucata         DSKISTTERVVKNVSFPPSHRLSMNEVFDA-KGKPRPDELKKHFILEGRVTEDVALRIINEGAALLRQEKTMIDIEAPVT 83
Haliotis discus         DGKLSTTERVVKSVPFPPSHRLTMSEVFDA-KGKPKPDVLKQHFILEGRVTEDVALRIINEGAALLRQEKTMIDIEAPVT 83
Drosophila melanogaster RSTISTKERVIDSVAFPPSRKLTCADVFDARTGKPQHDVLKQHFILEGRIEESAALRIIQEGATLLRTEKTMIDIEAPVT 160
Homo sapiens            DPKLSTTDRVVKAVPFPPSHRLTAKEVFDN-DGKPRVDILKAHLMKEGRLEESVALRIITEGASILRQEKNLLDIDAPVT 86

Crassostrea virginica   VCGDIHGQFYDLMKLFEVGGPPSTTRYLFLGDYVDRGYFSIECVLYLWALKILHPTTFFLLRGNHECRHLTEYFTFKQEC 163
Pinctada fucata         VCGDIHGQFYDLMKLFEVGGPPSTTRYLFLGDYVDRGYFSIECVLYIWALKILHPTTFFLLRGNHECRHLTEYFTFKQEC 163
Haliotis discus         VCGDIHGQFYDLMKLFEVGGPPATTRYLFLGDYVDRGYFSTECVLYLWALEILYPNTFFLLRGNHECRHLTEYFTFKQEC 163
Drosophila melanogaster VCGDIHGQFYDLMKLFEIGGSPATTKYLFLGDYVDRGYFSIECVLYLWSLKITYPQTLFLLRGNHECRHLTEYFTFKQEC 240
Homo sapiens            VCGDIHGQFFDLMKLFEVGGSPANTRYLFLGDYVDRGYFSIECVLYLWALKILYPKTLFLLRGNHECRHLTEYFTFKQEC 166

Crassostrea virginica   KIKYTEKVYDACMESFDCLPLAALMNQQFLCVHGGLSPEIHNLDDIRKLDRFKEPPAFGPMCDILWSDPLEDFGSERTSE 243
Pinctada fucata         KIKYTEKVYDACMEAFDCLPLAALMNQQFLCVHGGLSPEIHTLDDIRKLDRFKEPPAFGPMCDLLWSDPLEDFGTEKTSE 243
Haliotis discus         KIKYTERVYDACMESFDCLPLAALMNQQFLCVHGGLSPEIHNLGDIRKLDRFKEPPAFGPMCDILWSDPLEDFGNEKTTE 243
Drosophila melanogaster KIKYSERVYDACMDAFDCLPLAALMNQQFLCVHGGLSPEIHELEDIRRLDRFKEPPAFGPMCDLLWSDPLEDFGNEKNSD 320
Homo sapiens            KIKYSERVYDACMDAFDCLPLAALMNQQFLCVHGGLSPEINTLDDIRKLDRFKEPPAYGPMCDILWSDPLEDFGNEKTQE 246

Crassostrea virginica   HFTHNSVRGCSYFYSYAACCDFLQQNNLLSIIRAHEAQDAGYRMYRKSQTTGFPSLITIFSAPNYLDVYNNKAAILKYEN 323
Pinctada fucata         HFTHNSVRGCSYFYSYAACCDFLQQNNLLSIIRAHEAQDAGYRMYRKSQTTGFPSLITIFSAPNYLDVYNNKAAILKYEN 323
Haliotis discus         HFTHNTVRGCSYFYSYAACCDFLQQNNLLSIIRAHEAQDAGYRMYRKSQTTGFPSLITIFSAPNYLDVYNNKAAILKYEN 323
Drosophila melanogaster FYTHNSVRGCSYFYSYAACCDFLQNNNLLSIIRAHEAQDAGYRMYRKSQTTGFPSLITIFSAPNYLDVYNNKAAVLKYEN 400
Homo sapiens            HFTHNTVRGCSYFYSYPAVCEFLQHNNLLSILRAHEAQDAGYRMYRKSQTTGFPSLITIFSAPNYLDVYNNKAAVLKYEN 326

Crassostrea virginica   NVMNIRQFNCSPHPYWLPNFIDVFTWSLPFVGEKVTEMLVNVLSICSDDELIRDEDDTFEGSDEDGAVAARKEVIRNKIR 403
Pinctada fucata         NVMNIRQFNCSPHPYWLPNFMDVFTWSVPFVGEKVTEMLVNVLNICTDEELVTEEDEALEGTDEDGAVAARKEVIKNKIR 403
Haliotis discus         NVMNIRQFNCSPHPYWLPNFMDVFTWSLPFVGEKVTEMLVNILNICSDDELMTEGDDSL-------EVAARKEVIRNKIR 396
Drosophila melanogaster NVMNIRQFNCSPHPYWLPNFMDVFTWSLPFVGEKVTEMLVNVLNICSDDELMTEESEEPL---SDDEAALRKEVIRNKIR 477
Homo sapiens            NVMNIRQFNCSPHPYWLPNFMDVFTWSLPFVGEKVTEMLVNVLNICSDDELGSEED-GFDG----ATAAARKEVIRNKIR 401

Crassostrea virginica   AIGKMARVFTVLREESESVLQLKGLTPNGLLPLGALSGGKETLSTALSGFSPTHKISGFEEAKCLDKMNERRPPQKDALN 483
Pinctada fucata         AIGKMARVFTVLREESESVLQLKGLTPNGLLPLGALSGGKDTLKSALSGFSPSHKISGFEEAKCLDKMNERMPPRKDALN 483
Haliotis discus         AIGKMARVFTVLREESESVLQLKGLTPNGLLPLGALSGGKDTLKSALSGFSPTHKISGFEEAKCLDKLNERMPPRKDAVN 476
Drosophila melanogaster AIGKMARVFSVLREESESVLQLKGLTPTGALPLGALSGGKQSLKNAMQGFSPNHKITSFAEAKGLDAVNERMPPRRDATP 557
Homo sapiens            AIGKMARVFSVLREESESVLTLKGLTPTGMLPSGVLSGGKQTLQSAIKGFSPQHKITSFEEAKGLDRINERMPPRRDAMP 481

Crassostrea virginica   NSTR------------------------------ 487
Pinctada fucata         NSHS------------------------------ 487
Haliotis discus         QQNNKEKA-------------------------- 484
Drosophila melanogaster SPAEEGQKSLSAAAAAAANANANSING------- 584
Homo sapiens            SDANLNSINKALT----SETNGTDSNGSNSSNIQ 511
```

Supplementary Figure 9. Prediction of functional domains and 3D folding structures of *C. vriginica* calcineurin (CaN, Accession# XP_022317595). (A) Predicted protein phosphatase 2B (PP2B) domain in the C. virginica CaN (B) Predicted 3D structure of *C. virginica* CaN. Purple = PP2B domain. (C) Multiple amino acid sequence alignment of *C.virginica* CaN with other CaN across taxa, including *Pinctada fucata* (bivalve, Accession# ACI96106.1), *Haliotidis discus* (gastropod, Accession# ABO26624.1), *Drosophila melanogaster* (arthropod, Accession# NP_001245717.1), and *Homo sapiens* (vertebrate, Accession# NP_001124163.1). The methods used for protein 3D structure prediction and multiple alignment are described above in Supplementary Figure 8.

A

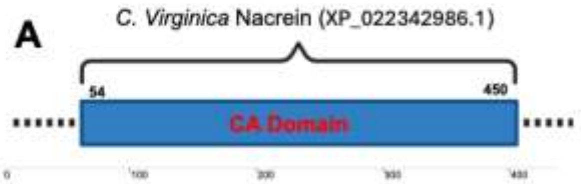

B

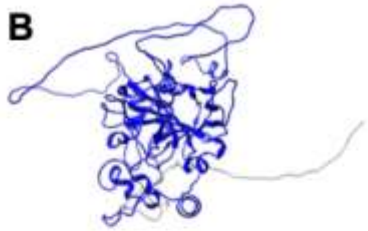

C

```
Crassostrea virginica   --MAVTVWLVFLGSLCVG--TVDSAGYL---GRLPHHEGQCYFEDI--NKAHFSYDRGSCREPENWCCLHKCWATCGSDE 71
Pinctada fucata         MFLLQLTLLAILGSHIRAVSIYKHRHYYKNHNGSPDDNGNCPDPKLSDCTAGFSYNREICPGPYDWSSISTCFHACNNGH 80
Magallana gigas         MKMAALYWLVFLGSLCIG--TVESAGYL---YRMPHHGDVCYYEDL--KNAHFSYDEENCRRPQEWCYLHKCWTTCGSTR 73
Mytilus edulis          --MELVFLFATVATVFVS--FVDGAGYL---QIRPPSANQCCYEDI--NQAHFSYDRDFCEGPKDWCRVHPCWTTCGSQR 71

Crassostrea virginica   RQSPRNIDTSGAKVWDL-P-LQILGKSS-RVPATVLNTGHSPHFTVKQD--KSNFILCLSHIHGTCPGDKYVFQDLHIHL 146
Pinctada fucata         RQSPINIDSDRAYYVPWLPRLHFESRYESEETEVSNHRNHAPEFESEDEE---EIYLK----LNRLRDEKYKFRNLHVHN 153
Magallana gigas         RQSPINLDTSEARRTKA-S-FQLYNKDK-RVPAFTYNNGHAPHFSVKLNETKRNITLKLKSVSGRNPNEEYILADLHIHL 150
Mytilus edulis          RQSPININTKETRYKRY-SRLKFGNLNT-RVLANIKNNGHAPDFDVDGKF---DDEITLCNVPRRPKDKKYNFAQLHVHL 146

Crassostrea virginica   GRDERKGSEHAIDGRHYPMEAHMVFYNSKYGNMS--------KAKSKADGLVVVGVMIQAHNRHKDDDDDTEEAKDEYER 218
Pinctada fucata         GNRKSKGSEHSVDGRFTPMEAHVVFYEDDDPSGQQASRVSIGGRYGYSDAFIVIGVFLEVDDDGFGDEPDDDECE-EILS 232
Magallana gigas         GKEKDVGSEHSIDGTFYPMEAHMVFYNSKYGDIS--------QAKPKDDGLVVIGVMIKAKRGHDDDDDDNN-------- 214
Mytilus edulis          GRDENKGSEHAIDNTFKPMEAHMVFYDSNYDGVG--------EAKPKRNGLVVLSVMIEVYGNNHEN------------- 205

Crassostrea virginica   GDDNERYDLEEHN-------------KH----EDADDANEEEEDEYERGDDNERSEWEKAHNNRYTYTRECCGRRQNG-- 279
Pinctada fucata         GDDYNKYKKNGKKGKYERRNTMDKSKIRQKFRNNYDDDDDGDDDNSSRSRSSRSSSSSESQENGYLDNNKYGWRWWNKY- 311
Magallana gigas         -----------------------------------DNDDEEEDDGYEKGDDKERSEWEAAWGHKYTYTRVCCHRNYGDYK 259
Mytilus edulis          -------------------------------------------------------------------------------- 205

Crassostrea virginica   ----DEGRPEKKCYKGSGKCKIRFARTLSYLMETYFEKVKEYHNGEKPEH--GGEGGNSTDKCDHEICNEKPSDEFIEQI 353
Pinctada fucata         -----------GDRGRRGRCKVRKARRLSRILECAYRNKKVHDFRHL--------------------------------- 347
Magallana gigas         HNGDGEDYFSKKCYKGNRKCKVRFARTLSHIMEKYYDKIKEYHHNEEEPK--E------IKDPEDLQCGEKPSDKTIQSE 331
Mytilus edulis          -----------NECRVDGTCKVRFAKKLSKLMEKYYEKVRHYPMESFNPDFFNLLGFQRSNSCTNNNCGRTPSPDFIEER 274

Crassostrea virginica   CQRKNNDESEYEVKVDCGISPYDVLPN-D-QRFYTYEGSLTTPPCYESVQWVMYKCPIRVSRRAFKALQAVKD-AEGHPI 430
Pinctada fucata         ---------DDNEVLEIDIRPEDVLPPPNHRQYYTYEGSLTTPPCNETVHWIVMKCHVKVSERVLHALRNVEGYPDGKTL 418
Magallana gigas         CTRGGGH-PEETYDIHCGISPVDVLPL-D-QRFYTYPGSLTTPPCYESVQWMVYKCPIKVSRKAFKALQHVED-AEKKPL 407
Mytilus edulis          CEKEEQN--ARSVRVTEGITPLDVLPY-DTNQFYTYAGSLTTPPCYETVQWVVYKCPIKVSSKAFRMLQLVED-SHFLPL 350

Crassostrea virginica   KEYGVRRPLQDNPKP-VFKNFIW 452
Pinctada fucata         SVFGTRRPTQYNENP-VYKNFWK 440
Magallana gigas         NHHGVKRPIKINEFFNVSKNF-- 428
Mytilus edulis          TRFGVRRPLQKNKKVVVYSNFKK 373
```

Supplementary Figure 10. Prediction of functional domains and 3D folding structures of *C. vriginica* Nacrein (Accession# XP_022317595). (A) Predicted carbonic anhydrase (CA) domain in *C. virginica* Nacrein. (B) Predicted 3D structure of *C. virginica* Nacrein. Blue = CA domain. (C) Multiple amino acid sequence alignment of *C.virginica* Nacrein with other putative Nacrein across diverse bivalve species, including *Pinctada fucata* (pearl oyster, Accession# AYN73059.1), *Magallana gigas* (Pacific oyster, Accession# NP_001292238.1), and *Mytilus edulis* (blue mussel, Accession# XP_071179650.1). The methods used for protein 3D structure prediction and multiple alignment are described above in Supplementary Figure 8.

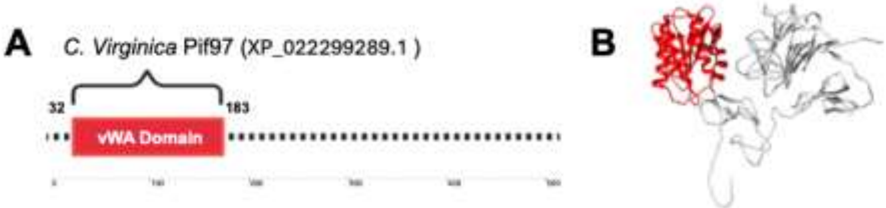

C

```
Crassostrea virginica    ------MRFVLCLFILL-SAVASFGQITPEAPKGCKELFDLAFVIDGSDSISAGDFETLRESISRMVDGFHIGSGETRMG 73
Pinctada fucata          -MQVPSIRVVFL-----LTAVFCVGVK----SDECK-TADVVVNVDASDDVSDQDFDKLKRAMLMMVRGLSIDDNQIRLG 69
Magallana gigas          ------MKVVPSLFIFIICVFISSGQRPDPFPKGCKAQIDVVFVIDGSDSISESDFQTLRTSISRIVDGFHIGSGETRMG 74
Mytilus edulis           MLKLPYMKVLFCSFIFLYSKVSSN----TIPLKGCQGKMDMIFVVDGSDHNTPSDFNILKDTLIELVERLHIGQGGARMG 76

Crassostrea virginica    IVVYSKDVAFTVPLSDDRVYLKNQARIMPHPREGTNTHLGIEAMTELFRKD-GRDGVPXGGVVVTDGIS-KESDKTLRFA 151
Pinctada fucata          MVTYGSEVCDSIPLQGDRLDLARTIRYMKKPTGPSKPFKGMGEARRMFSSR-GRYNVPHITMNLGGDIVDTEVKDLMDET 148
Magallana gigas          IIVYSKGVAFSVPLSYDPVYLKDQASIMPHPREGTNTHLGIEEMIDMFKKD-KRDGVPMAGVVVTDGIS-KEKEKTLLQS 152
Mytilus edulis           LIVFSQTVTKEITVTGNQALLKREITNLKQIGTGVNIATGLQTMIKMFTDTYKSVGVPKIGTVIATERS-TDPGQTIGVA 155

Crassostrea virginica    QIARDLGINMFSVGVGRYTEEKELIGIASAPNQAINVESFDELISILTKLVQLVCP------------------------ 207
Pinctada fucata          DKARDEDIKVMAIGLGAKVDRDEIESIAYDRDQAYFMDDEDDLIRKVKEIPDYLCKIIKAKKPKVSGGKKSKPAKKVDNG 228
Magallana gigas          RLARDLGINMFSVGVGRYTEEEELRGIASNPDQAIKVESFDELLKILSKLVQLVCP------------------------ 208
Mytilus edulis           FEAKNEGIRMNSIGVSTFADRTELSGIANADDKVLMVNTYQDLADAIGNVVTMVCPAQNLTTWIPP--------TTTTST 227

Crassostrea virginica    -------------------------------------------------------------------------------- 207
Pinctada fucata          PAGKSPGFDALKQSDDKSDKAKKVEVKELCDDAEWVDGVGYGSVPTRCEDFVMCQNV-SGSLRKTLKSCPFGQYWSKRQT 307
Magallana gigas          -------------------------------------------------------------------------------- 208
Mytilus edulis           TTTQAPE-------ITTPSRTKRSPISGPCEHCELRNGVGYNPHPEDCDKFIQCYFGENNEIVGAYRQCPWGQYWDQEVL 300

Crassostrea virginica    ------------NKCMMPGVVSYPHDISKNCRVYWKCEGDNSKLTCCPKVS-RTRPLSNLCTDPKCQDPC---GDE---- 267
Pinctada fucata          SCVLTEDEDCSDDLCKTMLLPSREYD--VSCRAYWKCEKGKSVARCCPSGMAY-EPGKGCVLDLDCDEECPPKNDGDDDD 384
Magallana gigas          ------------NKCMMPGVIAYPNDVSKNCRLYWKCEGEESKLTCCPRGFSFSAPVQSCIPDPKCVEPC---GDE---- 269
Mytilus edulis           TCRPAENVVCHKEKCRLPGLLSYPYGNIKNNRAYWKCNLGKSVSLCCPEGYLY-RPYQGCVEFNGKTETCPPIVFH---- 375

Crassostrea virginica    -------------GNSCNKRPVIYQPSKFEEFIPDYGWVQRACPPASVYDQVTCGCNIRPTLPPTP----PPPPRRVCQV 330
Pinctada fucata          DSSDEDDDDEIEYNPNCPLRPIKGHPEKFKQHTGDDNWEDFDCAPGTLFSARDCACSILGTAKKDDKNDDGGDAHKVCEP 464
Magallana gigas          -------------GPICNKRPSVYQPTIYEELIEGYGWVQRSCPPGTAYDRVTCGCTITQ-----T----PPPPRRVCRV 327
Mytilus edulis           -------------EGACDMRPVFGNATYYER-IVHGNWIRMPCAPGTHYDPTECACSLFYP----------TYISNECKP 431

Crassostrea virginica    LVHIPFDYD-CVDTSGNRFLIKNHGVKFT---RTGLALFENKAKLVIPNIQRY-LGSNFIIKMRYKEFPSF--------- 396
Pinctada fucata          ELYLPFCDD-LHDYSGKETHVENEGDAVI--IENGKAYFNGRAGLKIPRFSGVPYGKSVFIKMKYKEDEDDDKNKNDDDK 541
Magallana gigas          LVHIPFDID-CVDTSGNGFLIKNHGVKFT---RTGLALFEGKAKLVIPNIQRY-LGSNFLVKMRYKEFPSF--------- 393
Mytilus edulis           ELFISFDGTEIKDDSGKNNYIENHNVSIRKINGSSYGYFDGNSKIVIPRFANF-ESKDFVIKMNYSHTPND--------- 501

Crassostrea virginica    -------------------------------------------------------------------------------- 396
Pinctada fucata          KLRMKRDERSRKDYLKAILKRDDRKDKTDDTKGRRIIDRNDIIDDRRGRRKDDRKDGGRDDGKDGRRDDRKDNRLDDIKD 621
Magallana gigas          -------------------------------------------------------------------------------- 393
Mytilus edulis           -------------------------------------------------------------------------------- 501

Crassostrea virginica    ---EKQGLLSNGEC--------NSPTTLMVVKSSIRHTYKVQNAY---------------------G------------- 431
Pinctada fucata          KNDEPMTLISNGECDNFELNDCFEKPSIAITTGKKSAGFSVTSSEKDEVDLEIDEDKKGYLWDKEDGPDRNGKDKDRNGD 701
Magallana gigas          ---ETQGLLSNGDC--------YTPMTLQLIKDSIRHTYKIENSY---------------------R------------- 428
Mytilus edulis           ---RLESLISNGDDC------CDNVPSMALVKSKRNVHYMIKTVD----------------------DKT----------- 540

Crassostrea virginica    ---QN---------------------------SGF-------------------------S------------------- 437
Pinctada fucata          RSDDRRGYYWKKKDKDDNGKDKDKKGDKSDDKKGYYWKKDKDDKNGKDKDKRRDKSDDKKSLDDIVREIERSKGNGKDDN 781
Magallana gigas          ---QR---------------------------TGF-------------------------T------------------- 434
Mytilus edulis           REDSV---------------------------ATF-------------------------H------------------- 549

Crassostrea virginica    ---IPSSFQPWNEVTFSFADGFLAGSVNKVRNQKFTNGPIRRTPYGISIGFVDGFKNFQGYMDYVTIYKCGRANVYNKY- 513
Pinctada fucata          KDDEDDDKKGWKTVSLKISNGHIRGRRDDREDKDVLDGDLKTTFSGFQIGQGASNKNFKGYMDEVYIYFCDPGKEADYDD 861
Magallana gigas          ---IPTNFKPWNEVTFSHDGNTLAGSVNGIQNEKRSNGPIQRTPYGISIGFVDGYKHFQGYMDYVTIYKCANMNVYNKY- 510
Mytilus edulis           LPSIADPHNNWNEVYYIHDSLRLEGRSNGIRQEDFAVGKIKKTKAGLQIGAGRLFQNFTGLIDYVAVYLCRPSHL----- 624

Crassostrea virginica    -------------------------------------------------------------------------------- 513
Pinctada fucata          EDDDDDDDSDENDKNDDKKDGKKTDDKDKKDRKDGRDDRKDGRDDRKDRRDDRKDDRKGGKDDRKDDRKGGKDDRKDGR 941
Magallana gigas          -------------------------------------------------------------------------------- 510
Mytilus edulis           -------------------------------------------------------------------------------- 624

Crassostrea virginica    ------------------------------------------------------------------ 513
Pinctada fucata          DVRDDRDRGDKYDKKDDKDNDRLSDKDDRKDVDDNDKDDDNEKLYKRAMKKCDYVNKNVAKWLDKR 1007
Magallana gigas          ------------------------------------------------------------------ 510
Mytilus edulis           ------------------------------------------------------------------ 624
```

Supplementary Figure 11. Prediction of functional domains and 3D folding structures of *C. vriginica* Pif (Accession# XP_022299289.1). (A) Predicted Von Willebrand factor type A (vWA) domain in *C. virginica* Pif97 (B).Predicted 3D structure of *C. virginica* Pif. Red = vWA domain. (C) Multiple amino acid sequence alignment of *C.virginica* Pif with other putative Pif proteins across diverse bivalve species, including *Pinctada fucata* (pearl oyster, Accession# BAH97338.1), *Magallana gigas* (Pacific oyster, Accession# NP_001292217.1), and *Mytilus edulis* (blue mussel, Accession# XP_071120172.1). The methods used for protein 3D structure prediction and multiple alignment are described above in Supplementary Figure 8.

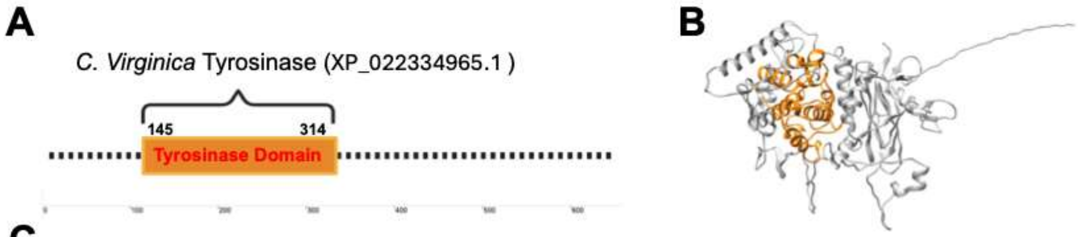

C

```
Crassostrea virginica    MRKGQTN-----LIVCFLATVVLPTSFGLIEEIQTPREILECLIYKTQNSSIGDISGRTVQDFCIRKYTLDTQSGKENFA 75
Pinctada fucata          MKMNLSNREV----VIFLLLAACTSAALLGDKYNVPPECMEEVIFDYDSPK----DNSTLNKDCVKFVSDSY--RKLQQL 70
Magallana gigas          MRKGQLN-----FIVCFLATVVLPTSFGLIEEIQTPRDILECLIYKSQNSTIGEVSGRTIQDFCIRKYTLDTQSGKENFA 75
Mytilus edulis           MTEKQMEAPTYVTLALLTLGYAVTVVVSLIEPVSLPYDLDRCITQHILKSNTSHTPPHFAEAFCVKKFILHNPNL--RVK 78

Crassostrea virginica    QNITTEGVQYLKSLFRQLESEIH---DQKRGKRQAGSWRVR-REIRTLSDAERNNVFQCFRRLKGDYSIDPRMSTYDLIS 151
Pinctada fucata          INGTDDDINYIRSLTREGMAL--LYPGSGREKRQAA--LRARRECRSLTSEEWRRLANAIRRLKFDPG-----NRFDTMA 141
Magallana gigas          KNISTEGVQYLKSLFRQLESEVH---DQKRGKRQAGTWRVR-REIRTLSDAERNNVFQCLRRLKSDYSIDPRMSTYDLIA 151
Mytilus edulis           VNFTTDETDYIQSLFKQVEADAGVDTDKGRFRRQTGGWRRHRREIRSAPYIEWQQYVRAVTRLKRVQSGTNGRSRYDTLA 158

Crassostrea virginica    SLHSGQA-ARLMHNGPAFLPRHMVYLLVMETACRVP-----IPYWDMTTDSEMMDPT--TSIVWSELFFGNGNGPVLTGP 223
Pinctada fucata          RIHAMPAVTANAHDGSSILGWHRVFLYLFENALRRKVPGVVLCYWDSTIDYLIPGPGQAQSSSFSHNMFGNSRGLVRTGP 221
Magallana gigas          SLHSGQA-ARMMHNGPAFLPRHMVYLLVMETACRVP-----MPYWDMTTDSEMMDPT--TSIVWSDLFFGPGNGPVLTGP 223
Mytilus edulis           AIHTAA--VDEVHFGPSFCPWHRLYLILVETALRVP-----IPYWDPTIDGAMDDPT--QSVVWTNKYFGNGNGPVVVGP 229

Crassostrea virginica    FAREFRTPIGTPIIRNIGSGGASLARKAGIRALLSRRRT---FEITEP-------QP-AQSIFSIEVHHNGVHNYIDGYMS 292
Pinctada fucata          FANEPTPWG-PLRRNFGGEGGSLMRPHVVDMIASDPRIRSHGQIVDGQGATGFIDSMTGQRTSLEAEHNNAHVAVGALMA 300
Magallana gigas          FGREFRTPTGTPIIRNIGSGGASLARKAGIRALLSRRRT---FEISEP-------QP-AQSIFSIEVHHNGVHNYIDGYMS 292
Mytilus edulis           FRHYYTPTG-PLSRDIGSDGLLFSKE-NIQRLFRFRFN---RDIFEP-------ASLGDQMSTLEGQHNMVHFWIGGHMN 297

Crassostrea virginica    GLNTASWDPVFWFIHSFFQLLWVAFRNGQ-RASGINPERDYPRGVRIPPGHEFYQRMNFMPFMRRITNLEGFSSRYDRI- 370
Pinctada fucata          VIPNAAWDPLFYFHHCYIDYVWQLFRRKLRNRLGIDPARDYLGHGGP----AHAPNAPLLGLIPGWRNVHGYSNVFTQRV 376
Magallana gigas          GLNTASWDPVFWFIHSFSQLLWVAFRNGQ-RANGINPERDYPRGVRVPAGHEFYQRMNFMPFMRRITNLEGLSNRYDRI- 370
Mytilus edulis           EFEYAAYDPVFWNYHAYVDYVWENFRRLQ-VRRGVNPEMDIVRSWQQDPDS-------FAVGLQGLRIREGYSGRIAGM- 368

Crassostrea virginica    VQYAPMPRCPA-CGGSP--YLACVRGVCVSRSSRRP--P-VFFRGKRSVSTPEDQLADANVNPNTTNLIQSNEAALSTLN 444
Pinctada fucata          YRYHFHPVCGNGCSGSTRRLLYCPG-----GGSRYRRCVSNTMPGRAQP-------------PALS---IAGRSAEEKFK 435
Magallana gigas          VQYAPMPRCPA-CGGSP--FLVCLRGVCVSRSSRRA--P-VFFRGKRSAGTSGDQLADENVNPNTTNLIQSNEAALSTLN 444
Mytilus edulis           VTYESMPKCPE-CGNSA--DMFCRRDVCVATSGSYRRNR-ISFPRRV--------FNRGSF-RNLFRRLRGKRSLISQFN 435

Crassostrea virginica    KPYQNTFMINGKIDEDAWAYIPIRVLYERPKGFNFHTTSPGATKRDMYDPENFKADAKRIGLHNEVLYKQQC--TPSGSG 522
Pinctada fucata          TVYDDPDIAS---------------------------------------------------------------------- 445
Magallana gigas          KPYQNTFMIDGKIDEDAWAYIPIRVLYERPKGFNFHTTSPGATKRDMYDPENFKNEAKRIGLHNEVQYKQQC--TPSGSG 522
Mytilus edulis           ------GVKDTSIELTDLAFVPVKVIRNRKEDITNYTSL-----------------NTTI--ETTQVVSQECIRHPETLH 490

Crassostrea virginica    AAKVFVQSNGLNYAGKYKDYAIVDERQPVTSAITYIGFKKPTDSDSDVILTAYDTCGRICRPACPVYGHHRESYKACSGS 602
Pinctada fucata          -------------------------------------------------------------------------------- 445
Magallana gigas          AAKVFVQSNGLNYAGKYKDYAIVDERQPVTSAITYVGFKKPSTSDSEVILTAYDTCGRICRPACPVYGHHRESYRACSGS 602
Mytilus edulis           NNIIYAESDGLNYKGKYKDFAYVNQ-----SAIIYIGAQKPTNDTSKVFITAYDRCGRICVPNCLVKDSQPPIYKICTGA 565

Crassostrea virginica    FRISSRSPLMFSQT-YGSAVSNTWNVREMEPGCQS-KSLPITFVCDHQNSWPWESKL 657
Pinctada fucata          --------------------------------------------------------- 445
Magallana gigas          FRISSKSPLMFGQN-YGSAVSSTWNVREMEPGCKSSQSLPITFVCDHQNSWPWESKL 658
Mytilus edulis           IRITSQNPVMYVSFPYDSELQQQ----------ANTAIPPIEFHCTF---------- 602
```

Supplementary Figure 12. Prediction of functional domains and 3D folding structures of *C. vriginica* Tyrosinase (Accession# XP_022317595). (A) Predicted tyrosinase domain in the C. virginica Tyrosinase Protein (B) Predicted 3D structure of *C. virginica* Tyrosinase. Orange = tyrosinase domain. (C) Multiple amino acid sequence alignment of *C.virginica* Tyrosinase with other putative tyrosinase proteins across diverse bivalve species, including *Pinctada fucata* (pearl oyster, Accession# AAZ66340.1), *Magallana gigas* (Pacific oyster, Accession# NP_001292226), and *Mytilus edulis* (blue mussel, Accession# XP_071175781). The methods used for protein 3D structure prediction and multiple alignment are described above in Supplementary Figure 8.

A

B

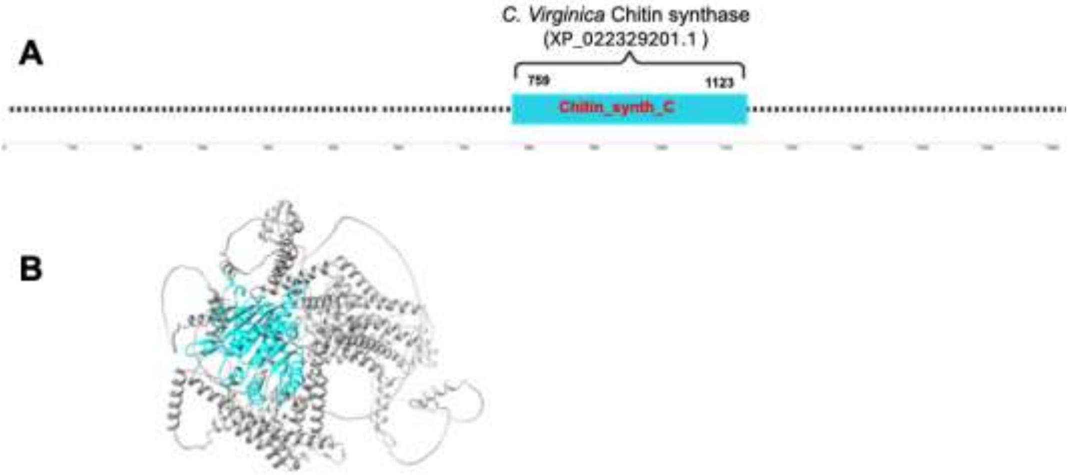

Supplementary Figure 13. Prediction of functional domains and 3D folding structures of C. vriginica Chitin Synthase (Accession# XP_022329201.1). (A) Predicted C-terminal domain in the C. virginica Chitin Synthase (Chitin_synth_C). (B) Predicted 3D structure of C. virginica Chitin Synthase. Cyan = Chitin_synth_C domain.

```
Crassostrea virginica   ------------------------MTAREDLTISMLLGDFVQRYRDAVYQPEQKLKLNRGNVESILREAGLTDYCLR-NQ 55
Pinctada fucata         LAANKFDSELVRRQLLCNGLMEIAELRRDGYPIRIKYEDFVERYRDISDFVNP-KSDDLGQTLVILKSLNIEGFKA-GRS 714
Magallana gigas         --------------------------MKEQFTVSMLLKDFIRRYRDISYQPDQIVGDNQENVECILQKAGLTGYNISGNE 54
Mytilus edulis          QVPDKLQTDLIVDQLKCNGLLEIARIRKNGYALRLTAEEFLQRYRDISFERKEIIQSTFDNCEKVLLDAKVTGYAF-GKQ 99
Atrina rigida           LSSGKFDSDLVRRQLLCNGLMEIAELRRDGYPIRIKFEDFAARYKDICDFGNT-NSDDLGKCLDILKTERIEGFKV-GRS 718

Crassostrea virginica   QVFIPQWHKSVLQELVFEKRKMAIVGQQHAVLPDSETDLDTTYDVLDESLPAA--RYVT--PTNA--------SSP-AVE 122
Pinctada fucata         KIFLKNWHGDILETKLREKQLENELRRRQS----EISLLSTMTDEEVESAPPTKADLHLSTDSGLGGSSNFFETKCKMS- 789
Magallana gigas         QVVIPQWHKSILQDLVYERRKMMVVGQQT-LLTDPETDLDTTFDVLDETLPNA--DFVE--STIK--------GQCFSGE 121
Mytilus edulis          KIFLRHWHGRILQDLVSEARTKMIEATGGAINDTA--------------IAET--EYIA--CSNTDGDDTVHEDTCDENS 161
Atrina rigida           KIFLKDYQKDMLEDTLREALRQKELERRRKA---EEEMLQAMREISDVDKHRQSTPLHGSADSGLVEDTDIYSQEL---- 791

Crassostrea virginica   MMKTETNISEGQSQ-------------IGKTHYGSQRKVTEWMQQNDDEKTEELT--------------------KPFIQ 169
Pinctada fucata         --HVDLGERMPILNN-HKKDTQSEVDTIDIDDSVSSPRKVS-KQSLDDGKS----------------------TEYTSME 843
Magallana gigas         TARTETNV--SSKN-------------VEKSQFGSQRKVTEWMQHIDCDTSEAQA--------------------KC--- 163
Mytilus edulis          TSKT-----TPKRS-------------NSCTSTSSQQRVAEWMQQNDNISEDVSLPMFEPGPPPVFPKPQRDQHVRISIP 223
Atrina rigida           ---HEMEHRIPIVDVDLSRDLESTADTIDIGDSVSVPKRSVYQGSADDGKS----------------------TNTTFID 846

Crassostrea virginica   ESPREKLLDVRLWDRFQMIPRERISTSDTFRPFLRVFKAICYALLFSIVLGSAVAAKTSILLMTSSIVKDENVRNVDNSP 249
Pinctada fucata         DIHEQDPHSWRPYDIFQISEREFEDSDYIFKEIMKGIRLFLYFFFFIMILGCAVTSKMSLLLTSGINKDTESRGEH--- 920
Magallana gigas         -NLQEKSLDARLWDRFQMIPRERVSTSDTFRPFLRLFKVLCYTLLFCIVLGSAVAAKTSILLMTSSIVKDENVRSVDNSP 242
Mytilus edulis          EYSERKVFDERLWDRFQMIPRERISTSDTIRPFVKVFKFICYVLLFCLVLTSAVVSKMCVLLLASSIIKDPVTKNDADSP 303
Atrina rigida           --QEVDSMQWRPYDIFQVAEREFEDQDYIFKEILKGIRLFLYVFFVIMIIGSIVASKMSLLLTSGINKDENSRGEH--- 921

Crassostrea virginica   THTMLLISICFPYVLWMLSYTAKSLFGSSAWPTLKTTTVTLFIELLHTFGLCLLVFHILPKTDMARSLLHLSGIFIIPAL 329
Pinctada fucata         -MVGLLFCMCGPMLWNWLMAFMKILFGGKEWPSFTTFAILLLLECLQTFGLCLLFRILPSTDFFRGLVISFAVCQIPSL 999
Magallana gigas         THTMLIISICFPYVLWVIGYTAKSLFGSCAWPSLKTTTVMLFIELLHSFGLCLMVFHILPKTDMARCLIHLSGIFVIPAF 322
Mytilus edulis          AYTMLVIAMCSPYICWVFSYSAKSLFGSSAWPSFRMVIVTLFVELLHTFGLSLLVFHILPRMDMARSLLTFCGVSTIPAF 383
Atrina rigida           -LVTLLFCMCGPMLWNWFMAFMRILFGGKEWPSMKTFLILLLFENVQTFGMCLLLFRVLPSTDFFRGLITTFAICQVPSL 1000

Crassostrea virginica   LRTTFVITDKSMSGIKRVTLTLVYGIAFLIQFCNLAATCMFSTSLTDGEKQLLVNQVIDDNDILTSINRPRPLFTNRIIW 409
Pinctada fucata         LKVIVHE-KRPNPSISEVVVIIMNIAAFLVQVSSIPF---FTV----GEFLKEGNHSIVEGYNQTTILRTSVTVPNNCEW 1071
Magallana gigas         LKTLFVITDKSLSGIKRVTLTLINGIAFLIQFGNLAATCMFSIPLTDGEKQSLVNDVIDNTHVITTIQRQRPLFTNRIIW 402
Mytilus edulis          FKTIGLACDSSIPPVKRCALGLINSLAFIIQLANLAASTLFSTPLTETEKNTLKATVLNNDKIVHTVSRPPLTFEDRIMW 463
Atrina rigida           LKVIVHE-KRPNPSVSEIVAIIMNISAFLVQVSAIPF---FSI----GEFMLQGNYSIVEGYNATTVTRTTVKLDSTCEW 1072

Crassostrea virginica   EIPVSLVFISLSFWENYAEGDVYI-CSFKIPLLEWKKNLIVVKQRLYVLVGLWKIGWTLVFAILLVPGLNFNMVFSDLNT 488
Pinctada fucata         ELPLAIILLSIGWWENYVSGEWTVFGRITIPFKQWRSILQDVRETSYFLIGPCKIGLCVLLSRLLTNNTNFILPATS--- 1148
Magallana gigas         EVPMALIFVSLSFWENYAEGDLYI-CSFKIPLLDWKKNLLLVKQRLYILIGLWKIGWTLIFAVLLVPGLNFNMNFSDLNS 481
Mytilus edulis          EIPVALLFISISYWENFVYGNFSI-NSFHIPLLEWKKQMHSVRQRLYIFAGIWKIGWTMVFAVLLFPGFNFNIHFSDKPQ 542
Atrina rigida           ELPVSLILLSIGWWENYVSGEWTVFGRITIPFKQWRSILQDVRETSYFLIAPFKIGLAVLMARLLTNNTPFVVPATG--- 1149

Crassostrea virginica   SETNRTGNDTERFNPLLT---SSRP----QNEISTIGTTTNFQINNLDPNNMERINLTKAIKDRGIESVLHLNDDMLDR 561
Pinctada fucata         -----------------EF-----------------NATTS---------------------------------------- 1155
Magallana gigas         VAGNHTKNNSTDLLPLAE---KK-------IDAPTIGITTSPLV---SGIDDFSQINLTKAIKDQGIKSVLHLTEEMLDR 548
Mytilus edulis          VVKSDNNVLPQMLTTTLSYKITESPTLRSISSLPT-KETTTPSIYDGSGRNIRVRRSIADDINTNGINAVTNLTSDTLNE 621
Atrina rigida           -----------------EF-----------------NATTS---------------------------------------- 1156

Crassostrea virginica   TQSV---------TPPKEN------VLLLKPENSTAN--NYLDAVKINFQKYGVLYLNIISGCLMSYFGSLACKLCMQIF 624
Pinctada fucata         ---------------------------------------KFSSKVEEVSVSYSLMFIQIGSGIICTYLAGLACKLHMQRM 1196
Magallana gigas         EQKV---------TGKNQN------ITNMKKNNTSNDYRYISEVIRLNFEKYGVLYLNIISNCLMAYFGSLACKLCMQLL 613
Mytilus edulis          KYQKLMSSTVSSITKSNTANNSTKPTAKSKTSNNNEFSFSIPEALKINFIQYGPLYLHLIASTLMSYFGSLACKLCMQTF 701
Atrina rigida           ---------------------------------------QYSSKAEEVGVAYSLMFIQIGSGIICSYLAGLACKLHMQRT 1197

Crassostrea virginica   GFTLPMFLATPVTLGLIFVQCYSQYIPSYLF--VWVCPEFKGDIRLYHLIWLGALWISQVITTSHIWFPRNGRMAKVERL 702
Pinctada fucata         AFALPLTLAPPLSLAVIYFQCEYHFLPAHWHMGGWFCPERSLMELLVPLICALLLWLSYCITVSHIWFPQSERMAKIEKL 1276
Magallana gigas         GFTVPMFLATPITFGLIIAQCNERLIPSYIY--VWVCPESKGDIRLFHLLWLGAIWISQIITTSHIWFPRNGRMAKVDRL 691
Mytilus edulis          GFTIPLFLATPATLGLIILAQSYTQFIPSFYF--VWICPEMEGNIRLYHLLWFGVLWISQVIVTAHIWSPKNGRMAKIDRL 779
Atrina rigida           AFALPLTLAPPVSLLFVYMQCEYNFLPSYWHMGGWFCPERDIISLLIPLICAVLLWLSYSITVSHIWFPQSERMAKIEKL 1277

Crassostrea virginica   FVTPIRCGIFADNSLALRRRLNDRDLSLFN-----NVDDWSYIYED-DDVYKADDVVPQIYACATMWHETRNEMIQLLKS 776
Pinctada fucata         FITPHFEGIFPDFTLGLRRRRNDKEVKLTGFDFTFRYVGEDTYYM---DDVYNGSNITPQVYVCATMWHETRQEMTQLLKS 1353
Magallana gigas         FVTPLRCGIFTDLSLTLRRRLNDRDFNLFN-----NEEECSYISED-DEIHKADDVVPQIYACATMWHETRNEMIQLLKS 765
Mytilus edulis          FVLPIRCGVLLDHSMMLRRRTNDRDCSLFN-----ADEDDSFIFDDDEEIHKADDVVPQIYACATMWHETRNEMTQLLKS 854
Atrina rigida           FITPHTDGILPDFTLSLRRRRNDKEVKLTGFDTFRYVGEDTYYM---DDIYSSSGVTPQVYACATMWHETRQEMTQLLKS 1354

Crassostrea virginica   LFRMDVDHSGRYLAQKFYGIRDPDYYEFEAHIFFDDAFDLTED-EVSVPNAFVWEFIDCIDDALSSVHERQLQLGPPIRT 855
Pinctada fucata         LFRLDYVHCASKLAQEKFRINDPDFFNLELHVIFDDAFELDEKVDKYIPNSFVKQLVECMEDAARSVVKGPISLLPPEKV 1433
Magallana gigas         IFRMDVDHSGRSLAQKFYCIRDPDYYEFEAHIFFDDAFDVPDDNHTSVPNEFVAEFVDCIDDALSSVHARQLQLGPPMRT 845
Mytilus edulis          IFRMDIDHSARFLAQKFYDIKDPDYYEFEAHIFFDDAMELSDD-DILVPNSFVADLIDCIEDALSSVHERQMYISSPVRT 933
Atrina rigida           LFRLDYVHCASRLAQEKFRIHDPDYYDLELHIIFDDAFELDDKVDKYVPNSFVRQLIECMEDAARSVVKGPISIQPPEKI 1434

Crassostrea virginica   ATPYGARLTWKLPGATVLVVHLKDRQQIRHKKRWSQVMYMYYLLGYRILAQPENLIQRNERNIVSTSEDTNRSMRLTNSQ 935
Pinctada fucata         ATPYGGKLIWTMPGHTKLVVHMKDKNKMRHRKRWSQCMYMYYLLGYKLFGAKEADNYMMEDAESSMTKLKNRKK-----G 1508
Magallana gigas         STPYGARLTWRLPGSTKLVVHLKDRQKIRHKKRWSQVMYMYYLLGYRILAQPENLLPRDERIFVSSSEETNRSTRLTSSQ 925
Mytilus edulis          PTPYGGRLIWRLPGGTKLIAHLKDKHKTRHKKRWSQVMYMYYLLGYRILAQPENLLRKDGDRTETPD-SDTSRVRLTETQ 1012
Atrina rigida           PTPYGGRLVWTMPGHTKLNVHMKDKNKMRHRKRWSQCMYLYYLLGYKLFGAREADRYMAEDAESSMTKVKNRKK-----S 1509

Crassostrea virginica   LRHRRQASHFTRSVIFNYTSEEVHTQAENTFILTLDGDVDFKPDAVRLLVDRLKKNKKVGAACGRIHPIGSGPIIWYQEF 1015
Pinctada fucata         KSKKTQRSRPLRSLFMR-MTPEQYEQADNTFILTLDGDVDFKPDSVKLLIDRMKKNKKVGAVCGRIHPIGSGPMVWYQQF 1587
Magallana gigas         LRHRRQASHFTRSVIFNYTSEEVHTQAENTFILTLDGDVEFKPDAVRLLVDRLKKNKKVGAACGRIHPIGSGPIVWYQEF 1005
```

```
Mytilus edulis          LRRRRQASHFTRSVIFNYTSEEVHTQAENTFILSLDGDVDFKPDAVRLLVDRLKKNKKVGAACGRIHPIGSGPILWYQEF 1092
Atrina rigida           KSKKCQRSRPLRSLFMR-MTPDQYEQAENTFMLTLDGDVDFRPDSVKLLIDRMKKNKKVGAVCGRIHPIGSGPMVWYQQF 1588

Crassostrea virginica   EYAIGHWLQKATEHVFGCVLCAPGCFSLFRGSALMDDNVARCYAIRASEAGHYVQYDQGEDRWLSTLLLQQGYRIDYCAA 1095
Pinctada fucata         EYAVGHWLQKAAEHVFGCVLCCPGCFSLFRGSAVMDDNVMKMYTTKPTEARHYIQFEQGEDRWLCTLMLQQGHRIDYCAG 1667
Magallana gigas         EYAIGHWLQKATEHVFGCVLCAPGCFSLFRGSALMDDNVARCYAIRASEAGHYVQYDQGEDRWLSTLLLQQGYRIDYCAA 1085
Mytilus edulis          EYAIGHWLQKATEHVFGCVLCAPGCFSLFRGSAIMDDNVARSYAIKTTEAGQYVQYDQGEDRWLSTLLLQQGYRIDYCAA 1172
Atrina rigida           EYAVGHWLQKAAEHVFGCVLCCPGCFSLFRGSAIMDDNVLKMYTTKPTEARHYIQFEQGEDRWLCTLMLQQGHRIDYCAG 1668

Crassostrea virginica   ADALTHAPETFSEFFNQRRRWGPSTLANLIDLLGDWKNTVRLNDNISTPYVLYQFFLLVSTVLGPATVLLMMAGAFTVVF 1175
Pinctada fucata         ADALTFAPETFNEFFNQRRRWSPSTLANMMDLLSSWRDTVRINDNISRPFVLYQFVLMASTILGPSTVILMITGSYHSVL 1747
Magallana gigas         SDALTHAPETFSEFFNQRRRWGPSTLANLIDLLGDWKNTVRLNDNISTPYVLYQFFLLVSTTLGPATVLLMMAGAFTVVF 1165
Mytilus edulis          ADALTHAPETFSEFFNQRRRWGPSTLANICDLLGDWKNTVRLNDNISTLYVLYQFLLLVSTVLGPATVLLMMAGAFTVVF 1252
Atrina rigida           ADALTFAPETFNEFFNQRRRWSPSTLANMMDLLSSWRDTVRINDNISRPYVLYQFVLMASTILGPSTIILMITGSYHSVL 1748

Crassostrea virginica   KTNVLESYAISLIPAVILIIVCMYAKPSTQIIVAIVASASYAIVMTIVLVGTVGTAIEGGLTSPNVVFLCMLVVIYFIAA 1255
Pinctada fucata         NLSIWQSYLLSLLPVVSYLVICMTMKSNHQITAAAVITALYSVIMMIATVGTIVSIVTENFGSPNVVFLSGLVIIFVIAG 1827
Magallana gigas         KTTVLESYVISLVPAIVLIVVCTYAKPSTQIIVATIASACYAIVMTIVLVGTVGTAIEGGLTSPNVVFLCMLVIIYFIAA 1245
Mytilus edulis          KTTIVESYAISLLPAIFFIVVCIFTKPTTQITVATIMSACYTIFMTIVLVGTIGTGIEGGITSPNVIFLMLLVIIFFISA 1332
Atrina rigida           GLNIWQSYLLSILPVMVYLAICMTMKSDHQIFAAAVITAIYSVVMMIATVGTIISIVTENFGSPNVVFLSGLVIIFVIAG 1828

Crassostrea virginica   LLHPEEFACVIPGALYFICIPTGYLVLTIYYLCNMHVVSWGTREVAFRKSESQIAEEEKAVEEKRKRKENKKGILGWLGI 1335
Pinctada fucata         ILHPQEFFCLVYGVLYFMTVPSTFILLTIYYLANLNNVSWGTRETPKKLTKEEEEEMKKMAEEK-KKKKESKSLLNRLGI 1906
Magallana gigas         LLHPEEFACVIPGALYFICIPSGYLVLTIYYLCNMHVVSWGTRETVSYKSKNQMDEEQKVLEEKSKTKKSKPGILGWLGI 1325
Mytilus edulis          LLHPEEFACLISGALYFICIPIGYLLLTIYYLCNLHVVSWGTREVPQRKSREEIEAEKRAEEEKRKKREERKGFLGWLGL 1412
Atrina rigida           ILHPQEFFCLIYGVLYFMTVPSTFVLLTVYYLCNLNNVSWGTRETPKKLTKEEEEELKLLQEEK-KKKKESKSLFNRLGI 1907

Crassostrea virginica   SYIFKEAVELFRQARALTL--DVQTKSKTDTLLEELISELRGKQTPNTENGKISES-TEPDT------------PISVKV 1400
Pinctada fucata         ININMNDFREVIRSLIGNSNAVEKKETTCSAVQTEN------------VMPPLERELSK---KSRHSEIKRPKEEE--VDV 1969
Magallana gigas         TSIFNEAVELFRQARSFSF--ESNSNSKTDTLLEELITELRNSHMPNVDDKEVCCN-TSVTKSKHSTVSQ---DSISTDI 1399
Mytilus edulis          DTVMNETVEMFKQFRQAASANKTDTKPKTDELLEELILELRQSRENKTLPPRQRSSSTMTDKGETPIVQVKRIEPVPGDM 1492
Atrina rigida           TSLISDARDLLKNILGTARNNERNMVSCA-VQTED------------TISPPERQLSR---HSRRSEENERQKEP--EDV 1969

Crassostrea virginica   ----ENDKESMPKQRTARTEEVLENPVFGEGPIKTLDEREKTFWKQMLQKYLFPIKEDKFHVEKMTSSLKNLRNNVVFGF 1476
Pinctada fucata         --------VPVGWEPDPLNPYWLKLDYLGNGPVSYIDQDETEFWRFMIKKYLHPLDEDQQHKQKIKEDLISLKNNVVFIY 2041
Magallana gigas         ----VKG-KDLRHSKRKNDDCLLENLVFGDGPIKHLEEREITFWKQMLQKYLYPIKEDKLHQLKMANSLKNLRNNVVFGF 1474
Mytilus edulis          SLIRTDSTPAWLRNEDPENPGWLTSSTCGEGPIKRLTEKERIFWKQLIKKYLFPINEDKMHQEKVAADLKNLRNNVVFGF 1572
Atrina rigida           --------VPQGWEPNPDHPYWLTMECFGNGPVSHIEHDEIDFWNFMIKKYLHPLDEDQQHKQKIKEDLICLKNNVVFIY 2041

Crassostrea virginica   FMTSALWIALTMQLQLLQDEFKNTILFIKIPHFYSSEKEMTFEPLGMIFLALFSAILFFQFMGMLSHRWGTILHVLSITN 1556
Pinctada fucata         CMINFLWTIITLQLQSMEDELKNFYII------------NKYEPLSLIFLSIFAIAISLQFLSMFMHRWGTFLHLMSSTR 2109
Magallana gigas         FMTSALWIALTMQLQLLQDDFKDTVLFIKIPHFYSSEKQMTFEPLGMIFLALFSAILLFQFLGMLSHRWGTILHVLSITD 1554
Mytilus edulis          FMTSALWVALTMQLQLLQDEFKDTVLFIKIPYFDSSRKDLTFEPLGMLFLALFTTILFFQFIGMLSHRWGTILHVLSITD 1652
Atrina rigida           FMINFLWTIITLQLQSMEDELKNFYII------------NKYEPLSLVFLSVFAIAITLQFLSMFIHRWGTFLHLMSSTR 2109

Crassostrea virginica   ISCSQKFAERYK-IQEVIAKVMELQRISNIENEPEPDYDDPIPDYEEDDFDEDDDETMYSFYSSSCSSTIN----TQANH 1631
Pinctada fucata         IDWLKKMHTEEDFVRFVVSEAQRLQR-----LEPEPDYDDLPPDYEDDETT--ATTPSE-QYDELPSLPAT----P---- 2173
Magallana gigas         IDCSQKFTEKYK-VQEVIAKAMELQRVCNIENEPEPDYDDPLPDYEDDDLEDDDETLS-TAYTSSSSS-TT----DHANQ 1627
Mytilus edulis          ISCTQQFTEKHK-IQEIITRTMELQRVINIENEPEPDYDDGLTDFDGEDFDDEDDISS-CFYTDTSSSSFSISRPPPSYH 1730
Atrina rigida           IDWFKKVHTEEDFVRFVVNEAQRLQR-----LEPEPDYDDLPPDYDDDGFTSSIETPSE-QYDELPSLPAS----P---- 2175

Crassostrea virginica   KNADTSKRASLRRRNSAVFNRRGLTTGRTLRRAFERRFRNELKREKSG-------------DQHSLSDFDH--------- 1689
Pinctada fucata         --RETFSRSVRRRYGT--MRDHENTDIPLLQQIFEDRLENIHRKWKRGTLAFRPNHLFTRTDSHRVSEKEQEMRQKMFKR 2249
Magallana gigas         QTSGVFKRSSLRRRNSAVFNRRGLTTGRTLRRAFERRYRYQLQKEKGV-------------DQHSESGFNH--------- 1685
Mytilus edulis          SHDITNGKKCIRRRHSAIFNRRGHATGRTLRHAFERRYRNELQRTTSG-------------DSENDTAAPE--------- 1788
Atrina rigida           --GATCRKISRSKSHKERSKNNFNKNVPVLQQIFENRLENIHRKWKQGTLAFRPNYRFDRTESHRFSEKEHIMRQKMFKR 2253

Crassostrea virginica   -------RKDEDL-NDGFLSV-------------- 1702
Pinctada fucata         SFRRIHSREDKSLEQN--------GQSIIIDMHKL 2276
Magallana gigas         -------LKDGEL-NDGFLSV-------------- 1698
Mytilus edulis          -------GAGNNIIRTASL---------------- 1800
Atrina rigida           SFRRINSKEDNKNHSDDFLDHRDMPQSIKIDMH—2286
```

Supplementary Figure 14. Multiple amino acid sequence alignment of *C.virginica* ChitinSynthase with other putative chitin synthase across diverse bivalve species, including *Pinctada fucata* (pearl oyster, Accession# BAF73720.1), *Magallana gigas* (pacific oyster, Accession# XP_011422034.3), *Mytilus edulis* (blue mussel, Accession# XP_071166727.1), and Atrina rigida (stiff penshell, Accession# AAY86556.1). The methods used for protein 3D structure prediction and multiple alignment are described above in Supplementary Figure 8.

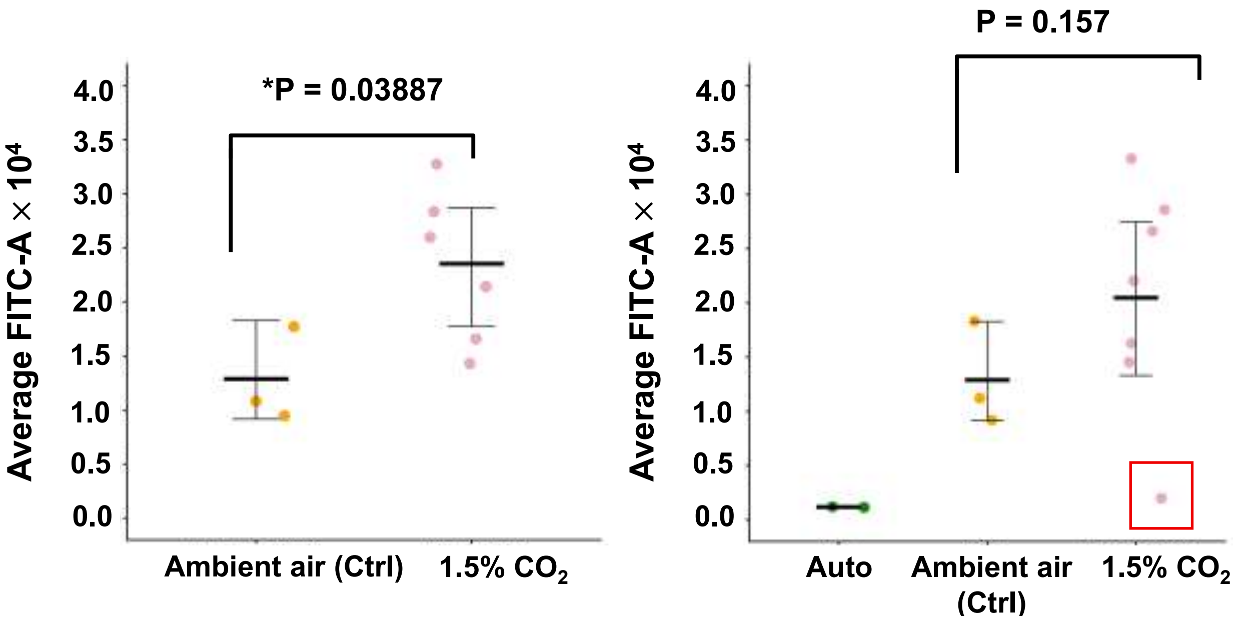

Supplementary Figure 15. Comparison of long-term intracellular calcium fluorescent signals (Fluo-4) in *C. virginica* mantle cells. (Left) Figure 1H provided in the manuscript with the technical outlier excluded. (Right) Figure 1H with the excluded data point retained (highlighted with a red outline). Auto: Water sample without Fluo-4 staining. Ambient air (Ctrl): Fluo-4 stained mantle cells under ambient air. 1.5% CO_2: Fluo-4 stained mantle cells treated with elevated1.5% CO_2. Note: The outlier value in the CO_2 treatment group is similar to the Auto group baseline value.

Supplementary References

1. Richards M, Xu W, Mallozzi A, Errera RM, Supan J. Production of Calcium-Binding Proteins in Crassostrea virginica in Response to Increased Environmental CO2 Concentration. Frontiers in Marine Science. 2018;5. Available: https://www.frontiersin.org/article/10.3389/fmars.2018.00203

2. Xue Q, Gauthier J, Schey K, Li Y, Cooper R, Anderson R, et al. Identification of a novel metal binding protein, segon, in plasma of the eastern oyster, *Crassostrea virginica*. Comparative Biochemistry and Physiology Part B: Biochemistry and Molecular Biology. 2012;163: 74–85. doi:10.1016/j.cbpb.2012.05.002

3. Li C, Huang J, Li S, Fan W, Hu Y, Wang Q, et al. Cloning, characterization and immunolocalization of two subunits of calcineurin from pearl oyster (Pinctada fucata). Comparative Biochemistry and Physiology Part B: Biochemistry and Molecular Biology. 2009;153: 43–53.

4. Miyamoto H, Miyashita T, Okushima M, Nakano S, Morita T, Matsushiro A. A carbonic anhydrase from the nacreous layer in oyster pearls. Proceedings of the National Academy of Sciences. 1996;93: 9657–9660. doi:10.1073/pnas.93.18.9657

5. Wang X, Song X, Wang T, Zhu Q, Miao G, Chen Y, et al. Evolution and functional analysis of the Pif97 gene of the Pacific oyster Crassostrea gigas. Current Zoology. 2013;59: 109–115.

6. Huan P, Liu G, Wang H, Liu B. Identification of a tyrosinase gene potentially involved in early larval shell biogenesis of the Pacific oyster Crassostrea gigas. Dev Genes Evol. 2013;223: 389–394. doi:10.1007/s00427-013-0450-z

7. Weiss IM, Schönitzer V, Eichner N, Sumper M. The chitin synthase involved in marine bivalve mollusk shell formation contains a myosin domain. FEBS Letters. 2006;580: 1846–1852. doi:10.1016/j.febslet.2006.02.044

8. Gattuso J-P, Alliouane S, Fischer P. High-frequency, year-round time series of the carbonate chemistry in a high-Arctic fjord (Svalbard). Earth System Science Data. 2023;15: 2809–2825.

9. Fujimura T, Wada K, Iwaki T. Development and morphology of the pearl oyster larvae, Pinctada fucata. Japanese Journal of Malacology (Japan). 1995.

10. Kurihara H, Kato S, Ishimatsu A. Effects of increased seawater pCO2 on early development of the oyster Crassostrea gigas. Aquatic Biology. 2007;1: 91–98. doi:10.3354/ab00009

11. Li H, Li Q, Yu H, Du S. Developmental dynamics of myogenesis in Pacific oyster Crassostrea gigas. Comparative Biochemistry and Physiology Part B: Biochemistry and Molecular Biology. 2019;227: 21–30. doi:10.1016/j.cbpb.2018.08.008

12. Stothard P. The sequence manipulation suite: JavaScript programs for analyzing and formatting protein and DNA sequences. Biotechniques. 2000;28: 1102–1104.
