# Supplementary material for: Ocean acidification disrupts the biomineralization process in the oyster Crassostrea virginica via intracellular calcium signaling dysregulation
Source: Commun Biol. 2026 Mar 17;9:607. doi: 10.1038/s42003-026-09861-y (PMC13144384; doi:10.1038/s42003-026-09861-y)
Supplement: Supplementary file 2 — Description of Additional Supplementary Files [file 42003_2026_9861_MOESM2_ESM.docx]

**Description of Additional Supplementary File**

File name: Supplementary Data 1
Description: This file contains the raw data supporting all findings reported in the manuscript. For each figure (Figures 1-6 and Supplementary Figures 1-14 and Tables 1-4), individual tabs in the spreadsheet provide the underlying numerical values. This includes raw measurements for all technical replicates or biological replicates for statistical analysis and plotting.
