# Supplementary material for: Ocean acidification disrupts the biomineralization process in the oyster Crassostrea virginica via intracellular calcium signaling dysregulation
Source: Commun Biol. 2026 Mar 17;9:607. doi: 10.1038/s42003-026-09861-y (PMC13144384; doi:10.1038/s42003-026-09861-y)
Supplement: Supplementary file 4 — Reporting Summary [file 42003_2026_9861_MOESM4_ESM.pdf]

nature portfolio

Corresponding author(s): Wei Xu

Last updated by author(s): Nov 18, 2025

Reporting Summary

Nature Portfolio wishes to improve the reproducibility of the work that we publish. This form provides structure for consistency and transparency in reporting. For further information on Nature Portfolio policies, see our Editorial Policies and the Editorial Policy Checklist.

Statistics

For all statistical analyses, confirm that the following items are present in the figure legend, table legend, main text, or Methods section.

n/a	Confirmed	
☐	☒	The exact sample size (*n*) for each experimental group/condition, given as a discrete number and unit of measurement
☐	☒	A statement on whether measurements were taken from distinct samples or whether the same sample was measured repeatedly
☐	☒	The statistical test(s) used AND whether they are one- or two-sided<br>*Only common tests should be described solely by name; describe more complex techniques in the Methods section.*
☒	☐	A description of all covariates tested
☐	☒	A description of any assumptions or corrections, such as tests of normality and adjustment for multiple comparisons
☐	☒	A full description of the statistical parameters including central tendency (e.g. means) or other basic estimates (e.g. regression coefficient) AND variation (e.g. standard deviation) or associated estimates of uncertainty (e.g. confidence intervals)
☐	☒	For null hypothesis testing, the test statistic (e.g. *F*, *t*, *r*) with confidence intervals, effect sizes, degrees of freedom and *P* value noted<br>*Give P values as exact values whenever suitable.*
☒	☐	For Bayesian analysis, information on the choice of priors and Markov chain Monte Carlo settings
☒	☐	For hierarchical and complex designs, identification of the appropriate level for tests and full reporting of outcomes
☒	☐	Estimates of effect sizes (e.g. Cohen's *d*, Pearson's *r*), indicating how they were calculated

Our web collection on statistics for biologists contains articles on many of the points above.

Software and code

Policy information about availability of computer code

Data collection	1. qPCR analysis: Performed using the QuantStudio 3 Real-Time PCR System (Applied Biosystems); 2. Immunofluorescence analysis: Fluorescent signal intensity was quantified with ImageJ, and images were acquired using an Olympus CKX53 microscope; 3. Oyster larvae size and shell organic matrix characterization: Data were acquired using an Olympus CKX53 microscope; 4. Calcineurin phosphatase activity assay: Conducted with a BIOTEK Cytation 5 image reader (Agilent); 5. Long-term calcium imaging analysis: Performed using the BD Accuri™ C6 Plus to detect calcium indicator signals
Data analysis	R version 3.6.2

For manuscripts utilizing custom algorithms or software that are central to the research but not yet described in published literature, software must be made available to editors and reviewers. We strongly encourage code deposition in a community repository (e.g. GitHub). See the Nature Portfolio guidelines for submitting code & software for further information.

Data

Policy information about availability of data

All manuscripts must include a data availability statement. This statement should provide the following information, where applicable:

- Accession codes, unique identifiers, or web links for publicly available datasets
- A description of any restrictions on data availability
- For clinical datasets or third party data, please ensure that the statement adheres to our policy

Provide your data availability statement here.

Research involving human participants, their data, or biological material

Policy information about studies with human participants or human data. See also policy information about sex, gender (identity/presentation), and sexual orientation and race, ethnicity and racism.

Reporting on sex and gender	This study does not involve human participants, and sex and gender were not considered in the study design.
Reporting on race, ethnicity, or other socially relevant groupings	This study did not report on race ethnicity, or other socially relevant groupings
Population characteristics	This study did not involve human participants, and no human population characteristics were reported.
Recruitment	This study did not involve human participants, and no recruitment process was conducted.
Ethics oversight	This study did not involve human participants.

Note that full information on the approval of the study protocol must also be provided in the manuscript.

Field-specific reporting

Please select the one below that is the best fit for your research. If you are not sure, read the appropriate sections before making your selection.

☒ Life sciences ☐ Behavioural & social sciences ☐ Ecological, evolutionary & environmental sciences

For a reference copy of the document with all sections, see nature.com/documents/nr-reporting-summary-flat.pdf

Life sciences study design

All studies must disclose on these points even when the disclosure is negative.

Sample size

1. Oyster used for primary cell culture were from 4-6 oysters for each treatment . The mantle cells from each oyster were isolated and seeded to 4-7 25 cm3 tissue culture flask. The RNA extraction were conducted from each well with highly viable (more than 50% confluence) cells. The number of oysters used in this study was based on our lab previous study: doi: 10.3389/fmars.2018.00203.
2. RNA extraction for in vivo oyster larvae were conducted by each beaker with around 500-10000 larvae depending on the developmental stages. The number of oyster larvae in each culturing beaker were estimated by a hemacytometer.

Data exclusions

1. qPCR data exclusions: qPCR reactions with Ct values greater than 35 were excluded from analysis, as amplification beyond this threshold is considered below the limit of reliable detection. Ct values above 35 are typically associated with very low template abundance and increased technical variability, making them indistinguishable from background signal. Besides, for each sample, qPCR was performed in triplicate to minimize technical errors, such as pipetting variability. Any replicate showing a standard deviation greater than 1 from the mean was excluded to avoid potential disturbance to the data.
2. Outliers were identified using the standard boxplot criterion of 1.5× the interquartile range (IQR). Data points falling outside this range were reviewed individually, and values were excluded only when a clear technical or experimental cause could be identified.

Replication

1. In vitro study: To verify reproducibility, mantle cells were independently isolated from oysters on multiple days. Experiments were conducted with these independently prepared samples, and the observed trends in transcriptional responses, protein expression levels, and calcineurin activities were consistent across all replicates. Variations in absolute values between preparations were observed, but the overall patterns and conclusions remained reproducible.
2. In vivo study: Oyster larvae used in this study were collected periodically from winter 2023 to summer 2024. For each collection, larvae were subjected to transcriptional analyses and shell matrix protein observations. Despite expected biological variation between batches, the overall trends under simulated ocean acidification and W-7 treatments were consistent across all independent preparations, confirming the reproducibility of the in vivo findings.
3. To ensure reproducibility, qPCR for each sample was performed in triplicate to minimize technical errors, such as pipetting variability. Any replicate with a standard deviation greater than 1 from the mean was excluded to prevent technical outliers from affecting the reproducibility of the results.
4. Immunofluorescence experiments were independently repeated on mantle cells prepared from differentoysters on different days, and for

each experiment, three images per well were captured for protein expression quantification. The overall trends were consistent across independent experiments.

Randomization	1. In vitro study: For each oyster, mantle explants were placed into 4-7 identical 25 cm3 tissue culture flaks (Thermo Fisher Scientific). To avoid allocation bias, flasks were randomly assigned to the control, elevated-CO2, or W-7 treatment groups by laboratory intern who were not involved in sample preparation or subsequent analyses. Interns selected flasks at random and placed them into different treatments. This flask-level randomization minimized systematic allocation bias and ensured an unbiased distribution of variability across treatments.<br>2. In vivo study: Larvae from each spawning batch were randomly collected by staff at the Texas A&M AgriLife Research Mariculture Facility who were not informed of the detail of this study. The larvae were evenly distributed into eight identical culture beakers. To prevent allocation bias, beakers were randomly placed to the ambient-air or elevated-CO2 glovebox by laboratory interns who were not involved in this study. Interns selected beakers randomly and placed four into the CO2 chamber and four into the ambient air environment. All beakers were maintained under identical culture condition except for the CO2 level. This beaker-level randomization could minize biased allocation of larval cultures across treatments.
Blinding	The experimenters maintaining the ocean acidification and W-7 treatments could not be blinded, as changes in pH caused the phenol red in the cell culture media to change color. However, data analysis was conducted using sample codes to ensure blinding to treatment groups.

Reporting for specific materials, systems and methods

We require information from authors about some types of materials, experimental systems and methods used in many studies. Here, indicate whether each material, system or method listed is relevant to your study. If you are not sure if a list item applies to your research, read the appropriate section before selecting a response.

Materials & experimental systems

n/a	Involved in the study
☐	☒ Antibodies
☒	☐ Eukaryotic cell lines
☒	☐ Palaeontology and archaeology
☐	☒ Animals and other organisms
☒	☐ Clinical data
☒	☐ Dual use research of concern
☒	☐ Plants

Methods

n/a	Involved in the study
☒	☐ ChIP-seq
☐	☒ Flow cytometry
☒	☐ MRI-based neuroimaging

Antibodies

Antibodies used	1. Calmodulin: mouse-anti-fungus calmodulin antibody (Uniprot ID: P02599, purchased from the Developmental Studies Hybridomas Bank at the University of Iowa, Iowa City, IA); 2. Calcineurin: mouse-anti human calcineurin-B primary antibody (Uniprot ID: O43745, purchased from the Developmental Studies Hybridomas Bank at theulin: mouse -anti-fungus calmodulin antibody (purchased from the Developmental Studies Hybridomas Bank at the University of Iowa, Iowa City, IA).
Validation	1. Calmodulin: Mouse anti-fungal calmodulin antibody (UniProt ID: P02599, https://dshb.biology.uiowa.edu/23-132-27?quantity=1&product-form=4) was used. Although this antibody was not specifically developed for oyster proteins, it effectively binds oyster calmodulin due to the high conservation of the calmodulin amino acid sequence across vertebrates and invertebrates.The antibody has been applied in study for cross-species applications (doi: 10.3389/fmars.2018.00203).<br><br>Calcineurin: Mouse anti-human calcineurin B antibody (UniProt ID: O43745, https://dshb.biology.uiowa.edu/CPTC-CHP2-1) was employed. Similar to calmodulin, this antibody was originally generated against human calcineurin B, but its binding to oyster calcineurin B is supported by the highly conserved amino acid sequence among diverse species. This Mouse anti-human calcineurin B antibody was first employed in the mollusk studies.<br><br>Notes: Both antibodies have been successfully applied in immunofluorescence experiments in oyster mantle cells, demonstrating specific staining signals consistent with the expected subcellular localization. Sequence alignment analyses confirm a high degree of homology with oyster proteins, providing a molecular rationale for their use despite the species difference.

Animals and other research organisms

Policy information about studies involving animals; ARRIVE guidelines recommended for reporting animal research, and Sex and Gender in Research

Laboratory animals	Eastern oyster: Crassostrea virginica south Texas strain
Wild animals	All oysters used in this study were farmed at the Texas A&M AgriLife Research Mariculture Facility and no wild oysters were used.
Reporting on sex	Sex was not considered in the study design
Field-collected samples	Adult C. virginica oysters of south Texas origin were obtained from the Texas A&M AgriLife Research Mariculture Facility and maintained in a recirculating system with 1 µm-filtered seawater from Corpus Christi Bay, Texas (~30ppt salinity) at 22~24 ∘C before

primary cell culture.
South Texas C. virginica oyster larvae were collected from the Texas A&M AgriLife Mariculture Center, following the center's spawning and culture protocols. A first generation of sexually mature female and male C. virginica, an inbred strain originally imported from Jaime J. Zapata Memorial Boat Ramp& Arturo Galvan Coastal Park, South Padre Island, TX, were dissected to collect eggs and sperm. Sperm from the males were rinsed into a beaker directly, and the activity were checked under the microscope. The egg suspension were filtered through a 44 µm nylon mesh and rinsed on a 20µm mesh. Sperm were added to egg suspension 30 minutes after egg collection, and about 5-10 sperm will surround an egg. The zygotes were incubated with 100 individuals/ml density in 5 litters of 100µm-filtered seawater at 23 °C. The salinity were around 33ppt. Within regular water change every two days, the larval density were changed to 20 individuals/ml on 2nd day, 10 individuals/ml on 7th day, 2.5 individuals/ml on 10th day, and 1.5 individuals/ml after the 14th day. After the insemination, the trochophore, D-shaped, umbonal (pre-metamorphosis), early pediveliger (post-eyed stage), and the spat stage were collected for ocean acidification treatment.

Ethics oversight

Oyster studies at Texas A&M University do not require IACUC approval. Guidance for oyster larvae culture was provided by the Texas A&M AgriLife Research Mariculture Facility (Corpus Christi, TX).

Note that full information on the approval of the study protocol must also be provided in the manuscript.

Plants

Seed stocks

This study did not involve plants.

Novel plant genotypes

This study did not involve plants.

Authentication

This study did not involve plants.

Flow Cytometry

Plots

Confirm that:

☒ The axis labels state the marker and fluorochrome used (e.g. CD4-FITC).

☒ The axis scales are clearly visible. Include numbers along axes only for bottom left plot of group (a 'group' is an analysis of identical markers).

☒ All plots are contour plots with outliers or pseudocolor plots.

☒ A numerical value for number of cells or percentage (with statistics) is provided.

Methodology

Sample preparation

Untreated and 24-hour 1.5% CO2-exposed mantle cells were incubated with Fluo-4 AM in oyster cell culture medium for 1 hour following the staining protocol above. After rinsing off the dye with DPBS, the stained mantle cells were incubated in fresh culture medium under ambient air or 1.5% CO2 for 24 hours. The treated oyster mantle cells were then harvested with 0.05% trypsin (Corning Inc.), and explants were removed using 40 µm cell strainers. Trypsinized mantle cells were rinsed and resuspended with DPBS for further flow cytometry analysis

Instrument

BD AccuriTM C6 Plus

Software

C6 Plus Analysis Software

Cell population abundance

After sorting, the abundance of events within each post-sort fraction was quantified by analyzing the full distribution of fluorescent signals (FITC-A), without pre-selecting a specific FSC/SSC gate. Purity of the sorted fractions was assessed by re-analyzing a sample of the sorted cells using the same fluorescence-based criteria. The proportion of events exhibiting fluorescence above the baseline (from unstained or negative controls) was reported as the fraction of Fluo-4–positive events. Because debris was included in the analysis, this measurement reflects the overall enrichment of fluorescent events in the post-sort fractions rather than the purity of intact epithelial cells specifically.

Gating strategy

Since no published gating strategy exists for oyster mantle epithelial cells, we first examined all events in the forward and side scatter (FSC/SSC) plots to characterize the overall cell population, including debris. Rather than applying a pre-defined FSC/SSC gate, we analyzed the full distribution of FITC-A fluorescence in fixed cell samples from both untreated and CO_2-treated groups to capture signals from intact cells and any debris. Regarding the background, disturbance we only excluded events detected in PBS-only samples from the analysis to ensure that only fluorescence originating from cellular material was measured. This approach allowed us to accurately quantify the total variation in intracellular calcium levels with flow cytometry reflecting the full cellular response beyond PBS background.

☐ Tick this box to confirm that a figure exemplifying the gating strategy is provided in the Supplementary Information.
